# Archaeal and bacterial diversity and community composition from 18 phylogenetically divergent sponge species in Vietnam

**DOI:** 10.7717/peerj.4970

**Published:** 2018-06-08

**Authors:** Ton That Huu Dat, Georg Steinert, Nguyen Thi Kim Cuc, Hauke Smidt, Detmer Sipkema

**Affiliations:** 1 Mientrung Institute for Scientific Research, Vietnam Academy of Science and Technology, Hanoi, Vietnam; 2 Laboratory of Microbiology, Wageningen University & Research, Wageningen, The Netherlands; 3 Institute of Marine Biochemistry, Vietnam Academy of Science and Technology, Ha Noi, Vietnam

**Keywords:** 16S rRNA, Prokaryotic diversity, Vietnam, Symbiosis, Porifera

## Abstract

Sponge-associated prokaryotic diversity has been studied from a wide range of marine environments across the globe. However, for certain regions, e.g., Vietnam, Thailand, Cambodia, and Singapore, an overview of the sponge-associated prokaryotic communities is still pending. In this study we characterized the prokaryotic communities from 27 specimens, comprising 18 marine sponge species, sampled from the central coastal region of Vietnam. Illumina MiSeq sequencing of 16S ribosomal RNA (rRNA) gene fragments was used to investigate sponge-associated bacterial and archaeal diversity. Overall, 14 bacterial phyla and one archaeal phylum were identified among all 27 samples. The phylum *Proteobacteria* was present in all sponges and the most prevalent phylum in 15 out of 18 sponge species, albeit with pronounced differences at the class level. In contrast, *Chloroflexi* was the most abundant phylum in *Halichondria* sp., whereas *Spirastrella* sp. and *Dactylospongia* sp. were dominated by *Actinobacteria*. Several bacterial phyla such as *Acidobacteria, Actinobacteria, Bacteroidetes, Chloroflexi, Deferribacteres, Gemmatimonadetes*, and *Nitrospirae* were found in two-thirds of the sponge species. Moreover, the phylum *Thaumarchaeota* (Archaea), which is known to comprise nitrifying archaea, was highly abundant among the majority of the 18 investigated sponge species. Altogether, this study demonstrates that the diversity of prokaryotic communities associated with Vietnamese sponges is comparable to sponge-prokaryotic assemblages from well-documented regions. Furthermore, the phylogenetically divergent sponges hosted species-specific prokaryotic communities, thus demonstrating the influence of host identity on the composition and diversity of the associated communities. Therefore, this high-throughput 16S rRNA gene amplicon analysis of Vietnamese sponge-prokaryotic communities provides a foundation for future studies on sponge symbiont function and sponge-derived bioactive compounds from this region.

## Introduction

Sponges (Porifera) are the sister group to all other metazoans (Simion et al., 2017), and are dated back at least 600 million years ago (Maloof et al., 2010; Yin et al., 2015). Marine sponges are distributed across a wide range of habitats, from polar regions, and temperate benthic communities to subtropical and tropical coral reefs (Van Soest et al., 2012). They play an important ecological role in the carbon, nitrogen, and sulfur cycles in benthic ecosystems, and both aerobic and anaerobic processes have been observed (Bell, 2008; Maldonado, Ribes & van Duyl, 2012). Moreover, the sponge microhabitat provides diverse niches for a wide range of different potential symbionts, including archaea, bacteria, microalgae, fungi, unicellular eukaryotes, as well as macrofaunal communities that can constitute a large fraction of the total sponge biomass (Bell, 2008; Duris et al., 2011; Lattig & Martín, 2011; Santavy, 1985; Santavy & Colwell, 1990; Sarà, 1971; Taylor et al., 2007; Thomas et al., 2016; Vacelet & Donadey, 1977; Wilkinson, 1978a, 1978b).

Due to the abundance and composition of the associated prokaryotes, most sponges can be grouped in either high microbial abundance (HMA) or low microbial abundance (LMA) species (Gloeckner et al., 2014; Moitinho-Silva et al., 2017b). HMA sponges contain a high concentration of microorganisms (10^8^–10^10^ microorganisms per gram sponge tissue), whereas the number of microorganisms in LMA sponges is much lower with only 10^5^–10^6^ microorganisms per gram sponge tissue (Hentschel, Usher & Taylor, 2006). In addition, LMA sponges harbor less diverse microbial communities at the phylum-level than HMA sponges (Bayer, Kamke & Hentschel, 2014; Moitinho-Silva et al., 2014; Schmitt et al., 2011). However, exceptions that deviate from this pattern have been reported (Easson & Thacker, 2014; Thomas et al., 2016). Several sponge microbiota studies revealed that sponge-associated prokaryotic communities are distinct from benthic and planktonic communities (Alex & Antunes, 2015; Naim et al., 2014; Rodríguez-Marconi et al., 2015; Weigel & Erwin, 2015), and that, besides evolutionary history of the sponge species, host identity is the most important factor for structuring the sponge-prokaryotic communities (Easson & Thacker, 2014; Steinert et al., 2016; Thomas et al., 2016). Previous studies report that sponge-associated prokaryotic communities are stable despite geographic differences and can exhibit monophyletic “sponge-specific” 16S ribosomal RNA (rRNA) gene sequences that are not present in the surrounding environments (Hentschel et al., 2002; Simister et al., 2012; Taylor et al., 2007). However, recent studies using deep sequencing revealed that some sequences in these monophyletic 16S RNA gene clusters are also found in seawater and sediment samples, albeit at very low abundances. Therefore, these clusters are not strictly “sponge-specific,” but better described as “sponge-enriched” (Taylor et al., 2013; Thomas et al., 2016). Although our knowledge about the prokaryotic communities associated with sponges has substantially improved in recent decades, our understanding about the ecological function of sponge symbionts is still limited. Animals typically excrete ammonium as inorganic metabolic waste rather than nitrate, thus the release of nitrate from sponges has been considered as sign for microbial nitrification in sponges (Corredor et al., 1988; Diaz & Ward, 1997; Jiménez & Ribes, 2007). Recent studies provided evidence of nitrification related to sponges symbionts based on incubation experiments as well as molecular markers (i.e., genes encoding for nitrification, for instance the *amoA* gene or *nxrB* gene) (Bayer, Schmitt & Hentschel, 2008; Diaz & Ward, 1997; Feng et al., 2016; Hoffmann et al., 2009; Steger et al., 2008). Studies investigating microbial nitrifiers in sponges reported many potential sponge symbionts that are related to nitrification such as *Nitrosopumilus* (AOA: ammonia-oxidizing archaea), *Nitrosococcus*, *Nitrosospira* (AOB: ammonia-oxidizing bacteria), and *Nitrospira* (NOB: nitrite-oxidizing bacteria) (Cuvelier et al., 2014; Feng et al., 2016; Hoffmann et al., 2009; Kennedy et al., 2014; Kowalchuk & Stephen, 2001; Pfister, Gilbert & Gibbons, 2014; Steger et al., 2008; Turque et al., 2010; Zhang et al., 2015).

Bacterial and archaeal diversity in sponges has been characterized from a wide range of marine regions across the globe including the Atlantic, Pacific, and Indian Oceans as well as the Mediterranean, Red Sea, the Caribbean, the Yellow Sea, and the South China Sea (Giles et al., 2013; Li et al., 2007, 2011; Thomas et al., 2016). Vietnam exhibits a high diversity of marine sponges with at least 229 species, belonging to 124 genera, 65 families, 18 orders, and four classes (Quang, 2013), however, sponge-associated prokaryotic communities from Vietnam still remain unexplored. Many sponges in Vietnam have been identified as potential sources for new bioactive compounds: new muurolane-type sesquiterpenes were isolated from the sponge *Dysidea cinerea* (Kiem et al., 2014), new anticancer sterols from an *Ianthella* sp. (Nguyen et al., 2009), antifouling 26,27-cyclosterols from *Xestospongia testudinaria* (Nguyen et al., 2013), and new sesquiterpenes and bis-sesquiterpene from *D. fragilis* (Cuc et al., 2015; Kiem et al., 2016; Nguyen et al., 2015). However, the true producers of many bioactive compounds from sponges are sponge-associated microorganisms rather than the sponges themselves (Fisch et al., 2009; Indraningrat, Smidt & Sipkema, 2016; Unson & Faulkner, 1993).

Therefore, we aimed to characterize the yet uninvestigated prokaryotic diversity and composition of Vietnamese sponges, and to provide a foundation for future studies focusing on sponge-associated bioactive compounds. We collected 27 sponge specimens, comprising 18 sponge species, from the central coastal region of Vietnam. Bacterial and archaeal diversity and sponge-specific community composition was characterized using Illumina MiSeq sequencing of PCR-amplified 16S rRNA gene fragments. To the best of our knowledge, among these 18 sponge species, six species (i.e., *Clathria reinwardti*, *Haliclona amboinensis*, *Cinachyrella schulzei*, *Haliclona fascigera*, *Tespios aploos*, and *Axos cliftoni*) are investigated for the first time regarding their prokaryotic communities.

## Materials and Methods

### Sampling

Sponge specimens (*n* = 27) were collected by scuba diving from May to September 2015 at three locations from the central coastal region of Vietnam at 5–10 m depth (Table S1). Lang Co Bay is located in the Phu Loc district within the Thua Thien Hue province. Con Co is a small island in the Quang Tri province with an area of 2.3 km^2^. Hon Mun Island is a marine conservation area in the Nha Trang Bay, Khanh Hoa province. Lang Co Bay was sampled in May 2015, Con Co Island in August 2015 and Hon Mun Island in September 2015. The distance between sampling sites ranges from 150 to 540 km. The specimens were transferred directly to zip-lock bags containing seawater to prevent contact of sponge tissue with air. All samples were immediately transported to the laboratory, rinsed three times with sterile artificial seawater (Instant Ocean Aquarium Sea Salt Mixture: Instant Ocean), and stored at −80 °C until DNA extraction.

### DNA extraction and PCR amplification of 16S rRNA genes

Sponge samples prior to extraction were processed using a modified protocol from (Abe et al., 2012). In brief, the specimens were rinsed three times with sterile artificial seawater to remove any debris attached to the sponge. Then the specimens were further cleaned with a sterile scalpel in order to remove any sediment and other organisms strictly attached to the sponge. Finally, a piece of sponge tissue was ground in TEN buffer (3.5% sodium chloride, 10 mM tris-hydroxymethyl-aminomethane, 50 mM ethylenediaminetetraacetic acid, pH 8.5) with a sterilized mortar and pestle. Cell suspensions were filtered through a large nylon mesh (20 μm) to remove potential contaminants. The filtrates were then centrifuged at 8,000 g for 15 min at 4 °C. The pellets were used to extract total genomic DNA using the ZymoBead^™^ Genomic DNA Kit (Zymo Research, Irvine, CA, USA) according to the manufacturer’s protocol. The concentration of the extracted DNA was determined with a Nanodrop 1000 spectrophotometer (Nanodrop Technologies, Wilmington, DE, USA), and its integrity was examined by gel electrophoresis on a 1% (w/v) agarose gel. The extracted DNA was dissolved in TE buffer and stored at −20 °C until further analysis.

The prokaryotic communities were characterized by Illumina MiSeq sequencing of 16S rRNA gene fragments using a two-step amplification procedure. The V4 region of the 16S rRNA genes was amplified by PCR using the 2nd version of the bacterial primers 515F/860R (Apprill et al., 2015), which were added to the 3′ end of Unitag1 and Unitag2, respectively (Tables S2 and S3). The PCR amplification was performed in a volume of 40 μL containing 8 μL of 5× Phusion HF green buffer (ThermoFisher Scientific, Waltham, MA, USA), 1 μL of a 10 mM dNTP mixture (Promega Benelux B.V., Leiden, The Netherlands), 0.4 μL of Phusion Hot Start II High-Fidelity DNA polymerase (2 U/μL; ThermoFisher Scientific, Waltham, MA, USA), 1 μL of a 10 μM solution of each primer, 1 μL template (20 ng/μL), and 27.6 μL nuclease free water. The PCR was performed using the following conditions: an initial denaturation at 98 °C for 30 s, followed by 25 cycles of denaturation at 98 °C for 5 s, annealing at 56 °C for 20 s, elongation at 72 °C for 20 s, and a final elongation at 72 °C for 5 min. Subsequently, the first PCR product was used as template in a second PCR in order to add sample specific barcodes (eight nucleotides). The second PCR consisted of 10 μL Phusion HF green buffer (ThermoFisher Scientific, Waltham, MA, USA), 1 μL dNTP mixture (Promega, Madison, WI, USA), 0.5 μL Phusion Hot Start II DNA polymerase (ThermoFisher Scientific, Waltham, MA, USA), 31 μL nuclease free water, 2.5 μL 10 μM forward primer (barcode-linker-Unitag1), 2.5 μL 10 μM reverse primer (barcode-linker-Unitag2) (Table S3) and 2.5 μL of the first PCR product. The PCR conditions were 98 °C for 30 s, followed by 5 cycles at 98 °C for 10 s, 52 °C for 20 s and 72 °C for 20 s, and final elongation at 72 °C for 10 min. All PCR products were analyzed on a 1.8% (w/v) agarose gel to verify the products. The PCR products were purified using the HighPrep^™^ PCR clean-up protocol-MagBio kit (Magbio, London, UK), and quantified using the Quant-iTdsDNA high-sensitivity assay kit and the Qubit fluorometer 2.0 (Invitrogen, Grand Island, NY, USA). Finally, samples were pooled in equimolar concentrations to ensure equal representation of each sample (including two mock communities as an internal standard to compare expected with observed 16S rRNA gene composition). The pooled library was purified, concentrated and quantified again with the HighPrep^™^ PCR clean-up kit (Magbio, London, UK) and the Quant-iTdsDNA high-sensitivity assay kit (Invitrogen, Grand Island, NY, USA). The samples were sequenced on the Genome Sequencer Illumina MiSeq at GATC Biotech, Germany. Sequencing data was deposited in the NCBI Sequence Read Archive under BioProject ID: PRJNA354731 with accession numbers: SRS1815731–SRS1815757 (Table S3).

### Sequence data analyses

Illumina sequencing data was processed and analyzed using the NG-Tax pipeline (Ramiro-Garcia et al., 2016). Briefly, paired-end libraries were combined, and only read pairs with perfectly matching primers and barcodes were retained. To this end, both primers were barcoded to facilitate identification of chimeras produced during library generation after pooling of individual PCR products. Both forward and reverse reads were trimmed to 100 bp to avoid overlap in forward and reverse reads, which would affect the quality filtering. Paired-end trimmed forward and reverse reads were concatenated to yield sequences of 200 bp that were used for subsequent sequence data processing. Demultiplexing, OTU picking, chimera removal and taxonomic assignment were performed within one single step using the *OTU_picking_pair_end_read* script in NG-Tax. Reads were ranked per sample by abundance and OTUs (at a 100% identity level) were added to an initial OTU table for that sample starting from the most abundant sequence until the abundance was lower than 0.1%. The final OTU table was created by clustering the reads that were initially discarded as they represented OTUs <0.1% of the relative abundance with the OTUs from the initial OTU table with a threshold of (98.5% similarity) (Ramiro-Garcia et al., 2016). Taxonomic assignment using the most abundant sequence of each OTU was done utilizing the UCLUST algorithm (Edgar, 2010) and the Silva 111 SSU Ref database (Yilmaz et al., 2014).

### Prokaryotic community analysis

Community composition summaries at phylum and class levels were created using the *summarize_taxa_through_plots.py* script from QIIME version 1.9.1 (Caporaso et al., 2010). Good’s coverage index, rarefaction curves, and alpha diversity metrics (e.g., Shannon, inverse Simpson, and evenness) were calculated using the QIIME script *alpha_rarefaction.py*. Alpha diversity indices calculated from prokaryotic communities of sponge-species with replicates were tested by the Kruskal–Wallis test using function *kruskal.test* within the FSA package (Ogle, 2017) of R v.3.3.1 (R Core Team, 2016). Heatmaps were generated for (a) prokaryotic composition at phylum and class level, (b) the most abundant OTUs containing at least 2.5% of the reads in at least one of the samples, (c) archaeal OTUs, and (d) OTUs related to known nitrifying taxa using the *heatmap.2* function of the gplots package in R (Warnes et al., 2016). For distance-based multivariate analyses, the 16S rRNA gene OTU table was standardized using the *decostand* function (method = “hellinger”) of the vegan package in R (Oksanen et al., 2016). Hierarchical cluster analysis was performed using the functions *vegdist* (method = “bray”) and *hclust* (method = “average”). The non-metric multidimensional scaling plot was created via the function *metaMDS* (Bray–Curtis distances) of the vegan package (Oksanen et al., 2016). Multivariate analysis based on Bray–Curtis dissimilarities of sponge-associated prokaryotic communities for sponge-species with replicates were performed using the functions *betadisper*, *permutest*, and the permutational multivariate analysis of variance (*adonis*) functions of the vegan package.

### Sponge-enriched OTUs

The 91 most abundant OTUs of our study (i.e., at least 2.5% of all reads in at least one of the samples) were used to identify OTUs which are significantly enriched in the 3,569 sponge specimens (comprising 269 sponge species) from the sponge microbiome project (Moitinho-Silva et al., 2017a). In brief, the representative sequences (*n* = 91) of the most abundant OTUs from our study were subjected to a BLAST search (Altschul et al., 1990) against a curated sponge microbiome database, containing 64,424 high quality deblurred subOTU sequences that were extracted from the sponge Earth Microbiome Project (EMP) database (https://github.com/amnona/SpongeEMP). The curated spongeEMP BLAST database and additional information describing the database creation can be accessed here: https://github.com/marinemoleco/spongeEMP_BLASTdb. The sponge microbiome project subOTU sequences with 100% similarity to the present 91 query sequences were uploaded to the spongeEMP online server (www.spongeemp.com) in order to identify OTUs that are significantly enriched in sponge EMP specimens (*p* < 0.05 for the category “host-associated” in the field env_package).

### Molecular sponge identification

For the taxonomic identification of the sponge specimens, near full length 18S rRNA gene and cytochrome *c* oxidase subunit I (COI) gene fragments from all samples were PCR amplified using the primer sets EukF/EukR (Medlin et al., 1988) for the 18S rRNA gene, and jgLCO1490/jgHCO2198 (Geller et al., 2013) for COI (Table S2). PCR products were cloned into pGEM-T Easy vector systems (Promega, Madison, WI, USA) according to the manufacturer’s protocol. Positive clones were selected and sequenced using the T7/SP6 primer pair. Sequences were trimmed (error probability limit 0.1) using Geneious 4.8.3 (Kearse et al., 2012). The cloning vector was identified using VecScreen (http://www.ncbi.nlm.nih.gov/tools/vecscreen/) and removed. For the 18S rRNA gene, forward and reverse sequences were assembled to obtain near full length fragments. The 18S rRNA sequences from this study and the three most similar sequences obtained through blasting against the NCBI nr/nt database were added to a set of existing sponge 18S rRNA sequences (Sipkema et al., 2009), and subsequently aligned using the MAFFT (v.7.222) program with the FFT-NS-i strategy (Katoh & Standley, 2013). The same strategy was followed for COI sequences obtained in this study. The additional three COI gene sequences were added based on the highest BLAST similarity to our sequences as obtained from the NCBI database (nr/nt database), and then aligned as described above. Phylogenetic trees for the 18S rRNA and COI genes were created using RAxML version 8.0.0 (Stamatakis, 2014) with the GTRGAMMA model and 1,000 bootstrap replicates. The final sponge taxonomy was determined based on the best position in both phylogenetic trees and their BLAST identity to sequences of sponge species deposited in the NCBI database. Specimens were identified to species level if their identities were at least 99% with sequences in the NCBI database. However, three species showed high identity (≥99%) with sequences from sponge species in the NCBI database, which known distribution typically does not include the Pacific Ocean according to the World Porifera Database (http://www.marinespecies.org/porifera/). For those samples, it was decided not to classify down to species level and maintain the generic status instead. All generated sequences were deposited in GenBank under the accession numbers: KX894454–KX894480 (18S rRNA genes) and KX894481–KX894504 (COI genes).

## Results

### Molecular sponge taxonomy

A total of 27 sponge specimens were collected from the central coastal region of Vietnam and identified by sequence analysis of 18S rRNA and COI taxonomic marker genes (Figs. S1 and S2). A total of 18 phylogenetically divergent sponge taxa were identified: *Axinyssa* sp., *X. testudinaria*, *C. reinwardti*, *Spirastrella* sp., *Dactylospongia* sp., *H. amboinensis*, *C. schulzei*, *Niphatidae* sp., *H. fascigera*, *Amphimedon* sp. 1, *Amphimedon* sp. 2, *Haplosclerida* sp., *Rhabdastrella globostellata*, *Spheciospongia* sp., *Halichondria* sp., *Tedania* sp., *T. aploos*, and *A. cliftoni* (Table S1).

### Sponge-associated prokaryotic communities

**Figure 1:**
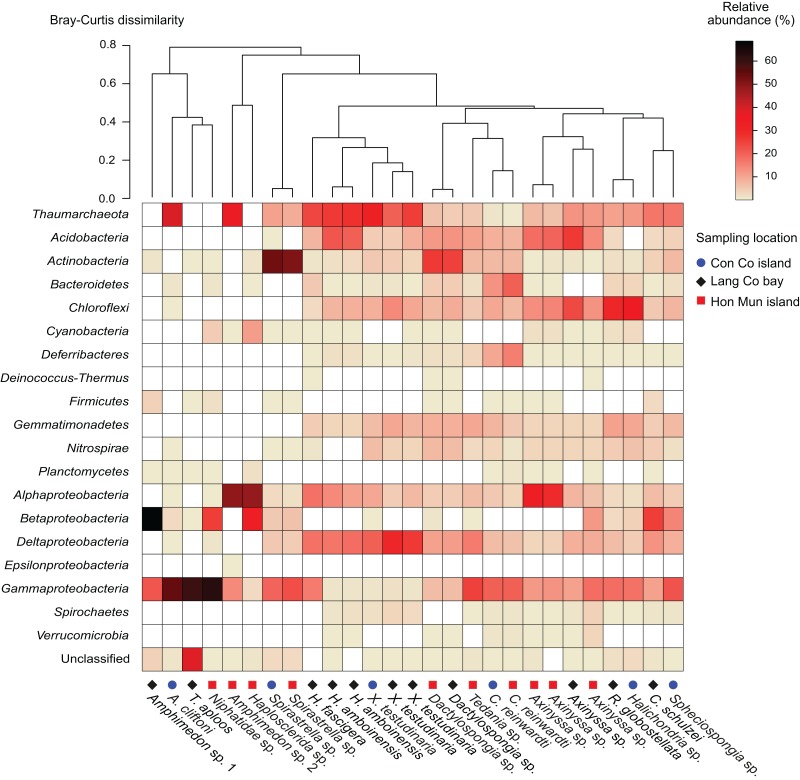
Heatmap of the prokaryotic composition and relative abundance of sponge-associated prokaryotes at phylum level (at class level for the phylum *Proteobacteria*). Samples were grouped using hierarchical clustering based on the Bray–Curtis distance matrix calculated from relative OTU abundances.

In total 5,326,187 high-quality reads were retained after quality filtering and were clustered into 926 OTUs. For all samples, rarefaction curves indicated near saturation, and coverage >99% (Table 1; Fig. S3). In addition, analysis of the bacterial profiles at the genus level showed a good correlation between observed and expected profiles (Pearson correlation coefficients were 0.7094 and 0.8308 for the two different mock communities). OTUs were classified into 14 bacterial phyla and one archaeal phylum (Fig. 1). Of all classified reads 88.2% belonged to the domain Bacteria, and 11.8% reads to the domain Archaea. Alpha diversity measures (i.e., Shannon, inverse Simpson, evenness) of the prokaryotic community associated with each specimen showed a wide range of diverse metric values. Evenness values ranged from 0.79 to 0.97, Shannon from 2.08 to 5.95, and inverse Simpson from 2.16 to 40 (Table 1). In our study, *T. aploos*, *Amphimedon* sp. 1, *Amphimedon* sp. 2, and *Niphatidae* sp. exhibited the lowest alpha diversity indices of the associated prokaryotic communities of all sponge species, whereas *Tedania* sp., *Dactylospongia* sp., and *C. reinwardti* exhibited the highest alpha diversity indices. The Kruskal–Wallis test for sponge species with duplicates showed significant differences among all prokaryotic alpha diversity indices (i.e., Shannon, inverse Simpson, OTU richness, evenness) (Table S4).

**Table 1:** Sequence statistics and alpha diversity of Vietnamese sponge-associated prokaryotic communities, including the total number of OTUs, coverage, and alpha diversity metrics.

Sample	Taxon	No. of OTUs	Coverage	Evenness	Shannon	Inverse Simpson
AMC	*Amphimedon* sp. 1	47	99.8	0.87	2.25	2.16
AMQ	*Amphimedon* sp. 2	31	99.8	0.84	2.14	2.90
AXT.1	*Axinyssa* sp.	94	99.6	0.94	4.95	18.52
AXT.2	*Axinyssa* sp.	107	99.5	0.94	4.98	17.86
AXT.3	*Axinyssa* sp.	85	99.6	0.94	4.70	15.15
AXT.4	*Axinyssa* sp.	99	99.6	0.94	4.84	15.87
AXC	*Axos cliftoni*	92	99.7	0.95	4.56	10.64
CIS	*Cinachyrella schulzei*	115	99.4	0.95	4.87	10.75
CLR.1	*Clathria reinwardti*	145	99.3	0.96	5.84	27.03
CLR.2	*Clathria reinwardti*	176	99.5	0.97	5.95	38.46
DAS.1	*Dactylospongia* sp.	157	99.2	0.96	5.81	30.30
DAS.2	*Dactylospongia* sp.	141	99.4	0.96	5.70	29.41
HAS	*Halichondria* sp.	108	99.5	0.94	4.63	11.24
HAA.1	*Haliclona amboinensis*	118	99.5	0.93	4.62	10.99
HAA.2	*Haliclona amboinensis*	92	99.5	0.93	4.33	9.26
HAF	*Haliclona fascigera*	89	99.6	0.93	4.77	15.39
CRV	*Haplosclerida* sp.	58	99.8	0.90	3.58	6.80
NIS	*Niphatidae* sp.	35	99.8	0.86	2.08	2.36
RHG	*Rhabdastrella globostellata*	146	99.3	0.96	5.36	15.63
SPV	*Spheciospongia* sp.	122	99.2	0.96	5.66	26.31
SPS.1	*Spirastrella* sp.	98	99.6	0.93	4.37	9.71
SPS.2	*Spirastrella* sp.	76	99.7	0.92	3.93	7.94
TES	*Tedania* sp.	165	99.9	0.97	6.04	40.00
TEA	*Terpios aploos*	19	99.9	0.79	2.12	3.47
XES.1	*Xestospongia testudinaria*	92	99.6	0.95	4.86	13.16
XES.2	*Xestospongia testudinaria*	121	99.6	0.95	4.96	12.35
XES.3	*Xestospongia testudinaria*	117	99.5	0.95	5.02	16.39

**Note:**

Alpha diversity values (evenness, Shannon, inverse Simpson) were calculated based on subsampling size of the sample with the fewest sequences (*n* = 1,595 reads).

*Proteobacteria* was the most abundant taxon in 15 of the 18 sponge species, making up 23.6% (*Dactylospongia* sp.) to 90.5% (*Amphimedon* sp. 1) of the total community in a sample. Although the phylum *Proteobacteria* was predominant in most sponge species, individual sponge species hosted different abundant proteobacterial classes: *Alpha-*, *Beta-*, and *Gammaproteobacteria* (Fig. 1). In contrast, *Chloroflexi* was the most abundant phylum in *Halichondria* sp., whereas *Spirastrella* sp. and *Dactylospongia* sp. were dominated by *Actinobacteria*. Other major phyla such as *Acidobacteria, Actinobacteria, Chloroflexi*, and *Gemmatimonadetes* were present at high relative abundances in different sponges (on average 4.5–8.7% for all samples). Remaining phyla (e.g., *Nitrospirae*, *Bacteroidetes*, *Cyanobacteria*, *Deferribacteres, Spirochaetes*) were detected at a lower relative abundance (<2.5%).

In this study, all archaeal OTUs (*n* = 73) belonged to the phylum *Thaumarchaeota* (Fig. S4). The *Thaumarchaeota* were found in a wide range of species (14 out of 18 species as well as all specimens of the same species if replicates were available) with relative abundances ranging from 0.4% (*C. reinwardti*) to almost 40% (*A. cliftoni*), with the majority of samples exhibiting relative abundances of archaea greater than 10%. Most archaeal OTUs were assigned to Marine Group I (*n* = 68), and only five OTUs were assigned to the Soil Crenarchaeotic Group (SCG). The OTUs belonging to Marine Group I were found in all the 14 species that contained archaea, whereas the OTUs belonging to SCG were found in only two species (Fig. S4).

**Figure 2:**
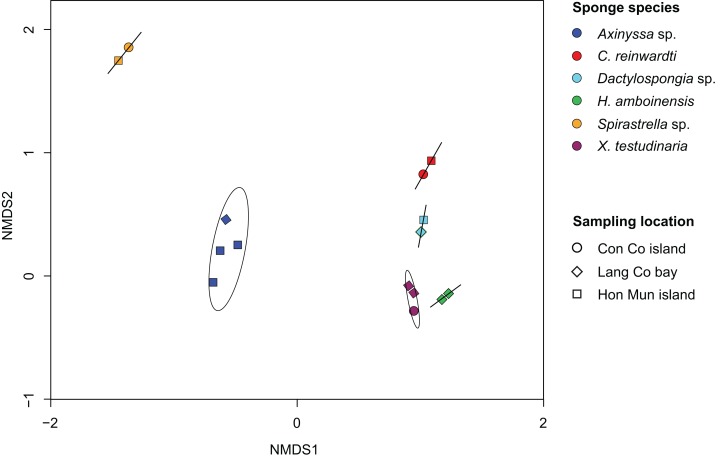
Non-metric multidimensional scaling (NMDS) plot derived from Bray–Curtis distances of sponge prokaryotic communities at OTUs level, NMDS stress value = 0.116. The samples of the same species were grouped with ordination ellipse using function *ordiellipse* of vegan package.

Non-metric multidimensional analysis of the sponge-associated prokaryotic community based on Bray–Curtis dissimilarity showed that replicate specimens of a species (i.e., *Axinyssa* sp., *C. reinwardti*, *Dactylospongia* sp., *H. amboinensis*, *Spirastrella* sp., and *X. testudinaria*) clustered together (Fig. 2). The analysis using adonis based on Bray–Curtis dissimilarity showed strong support for the effect of host-identity on their prokaryotic communities for these species (*R^2^* = 0.94, *p* < 0.001) (Table S5). This was further supported by hierarchical clustering with sponge samples belonging to the same species clustering together in spite of different sampling locations (Fig. S5).

### Analysis of the most abundant OTUs

**Figure 3:**
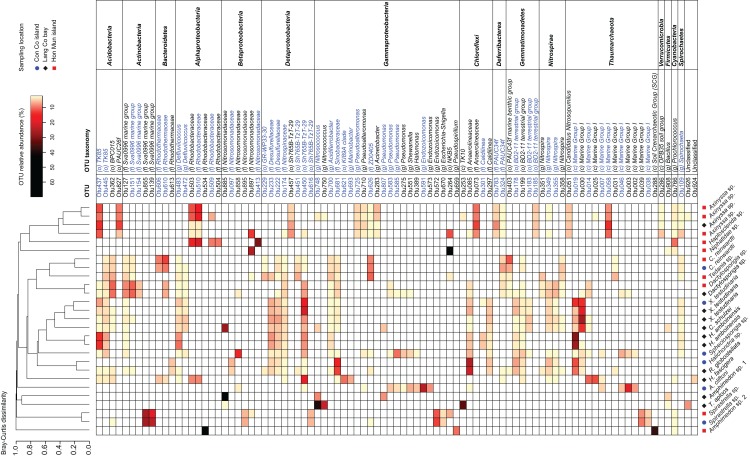
Heatmap of the most abundant OTUs (>=2.5% of the total reads in at least one of the samples). Samples were grouped using hierarchical clustering based on the Bray–Curtis distance matrix calculated from the relative abundances of these OTUs. If applicable, OTU taxonomy was assigned to the phylum (p), class (c), order (o), family (f), or genus (g) by NG-tax. The blue colors of OTU names indicate that these significantly sponge-enriched OTUs in the sponge microbiome project database (http://www.spongeemp.com).

The 91 OTUs with a relative abundance of at least 2.5% in one of the samples belong to 13 different phyla (Fig. 3). Among these 13 phyla, the following families and genera were most prominent: *Rhodobacteraceae*, *Nitrosomonadaceae, Sva0996 marine group, Nitrosococcus*, and *Caldilinea*. Approximately two-thirds of these 91 OTUs were shared at least among two sponge species (Fig. 3). These OTUs belonged to prokaryotic taxa such as *TK85*, *Sva0996 marine group*, *Defluviicoccus*, *Desulfurellaceae*, *Nitrospinaceae*, *Sh765B-TzT-29*, *BD2-11*, *Nitrospira*, *Marine Group I*. Furthermore, 75 out of the 91 OTUs are 100% similar to OTUs present in the sponge microbiome database, of which 45 OTUs were significantly enriched in the sponge microbiome project specimens (Fig. 3). The significantly enriched OTUs were mainly found in *Actinobacteria* (*Sva0996 marine group*), *Bacteroidetes* (*Rhodothermaceae*), *Alphaproteobacteria* (*Defluviicoccus, Rhodobacteraceae*), *Deltaproteobacteria* (*Desulfurellales*, *Sh765B-TzT-29*), *Gammaproteobacteria* (*Pseudomonas), Deferribacteres* (*PAUC34f*), *Gemmatimonadetes* (*BD2-11 terrestrial group), Nitrospirae* (*Nitrospira*), *Thaumarchaeota* (*Marine group I*).

## Discussion

### Microbial communities in Vietnamese marine sponges

To the best of our knowledge, of the 18 sponge species in the present study, the associated prokaryotic communities were examined for the first time for the following six species: *C. reinwardti*, *C. schulzei*, *H. amboinensis*, *H. fascigera*, *A. cliftoni*, and *T. aploos.* Moreover, this is the first time that the associated prokaryotic community of a sponge from the genus *Axos* has been described. All 15 prokaryotic phyla detected belong to the 41 phyla that have so far been detected in marine sponges (Thomas et al., 2016). The prokaryotic taxa with a high relative abundance in the present study (e.g., *Nitrospinaceae, PAUC34f, Caldilineaceae*, *Nitrosomonadales*, *Rhodobacteraceae*, *Endozoicomonas*, *Rhodospirillaceae*) are also abundant in other marine sponges, which can be found in vastly different marine regions (Rodríguez-Marconi et al., 2015; Steinert et al., 2016; Thomas et al., 2016). Regarding the six newly studied sponges, in four of them (i.e., *C. reinwardti, H. amboinensis, H. fascigera, C. schulzei*) all prokaryotic phyla were also found in other sponge species belonging to the same sponge genera (Alex & Antunes, 2015; Cleary et al., 2013; Erwin, Olson & Thacker, 2011; Jasmin, Anas & Nair, 2015; Khan et al., 2013; Naim et al., 2014; Sipkema et al., 2009; Thomas et al., 2016). In contrast, three additional bacterial phyla (i.e., *Actinobacteria, Firmicutes, Planctomycetes*) have not been found to be associated with the genus *Terpios* in a previous study (Tang et al., 2011).

Furthermore, we observed that members of the *Thaumarchaeota* were associated with a wide range of Vietnamese sponges and that they accounted for a high relative abundance (>10%) in over half of the prokaryotic communities studied here (15 out of 27 samples). This phylum has also been detected in many other sponge taxa from the Pacific, Atlantic, Antarctic ocean, Mediterranean Sea, Caribbean, and Floridian reefs as well as in cold-water sponges (Alex & Antunes, 2015; Cuvelier et al., 2014; Dupont et al., 2013; Lee et al., 2011; Margot et al., 2002; Polónia et al., 2014; Radax et al., 2012; Rodríguez-Marconi et al., 2015; Webster, Watts & Hill, 2001) with particular high abundances in deep-sea sponges (Jackson et al., 2013; Kennedy et al., 2014). Members of the *Thaumarchaeota*—formerly Marine Group I *Crenarchaeota*—are capable of oxidizing ammonia and play an important role in the nitrogen cycle in marine environments (Capone et al., 2008; Prosser & Nicol, 2008; Ward, 2011). These AOA are the primary ammonia-oxidizing components in marine systems, displaying *amoA* abundances of up to 10^8^ copies L^−1^ (Tolar, King & Hollibaugh, 2013; Wuchter et al., 2006). It has been reported that nitrogen input such as ammonia is one of the factors influencing the abundance of *Thaumarchaeota* (Hatzenpichler, 2012; Herfort et al., 2007; Hong & Cho, 2015; Kirchman et al., 2007; Oton et al., 2015). The environmental monitoring data at the sampling locations from our study revealed varying concentrations of inorganic nitrogen. For example, the ammonia concentration varied from 0.05 to 0.3 mg/L in Quang Tri, to 0.03–0.1 mg/L in Lang Co bay and was lowest (0.003–0.008 mg/L) in Hon Mun island as this island is in a protected area (Linh et al., 2015; MONRE, 2016; QCEEM, 2015). However, besides nitrogen input, other environmental factors may influence the abundance of *Thaumarchaeota* lineages, such as pH, depth, oxygen levels, as well as other organic substrates (Di et al., 2010; Hong & Cho, 2015; Isobe et al., 2012; Tolar, King & Hollibaugh, 2013; Verhamme, Prosser & Nicol, 2011; Yao et al., 2013).

Finally, Bray–Curtis dissimilarity analysis of the prokaryotic communities in those sponge species with replicates (i.e., *Axinyssa* sp., *C. reinwardti*, *Dactylospongia* sp., *H. amboinensis*, *Spirastrella* sp., and *X. testudinaria*) showed a similar pattern of host-specificity, which was already found in other studies (Easson & Thacker, 2014; Naim et al., 2014; Pita et al., 2013; Steinert et al., 2016; Thomas et al., 2016). In addition, specimens sampled from different locations at different time points still clustered together, supporting earlier observations of stable host-specific prokaryotic communities despite geographic and temporal differences (Erwin et al., 2015; Hardoim & Costa, 2014; Pita et al., 2013; Taylor et al., 2007).

### Sponge-enriched OTUs

Previous studies have identified “sponge-specific” and/or “sponge coral-specific” clusters based on monophyletic 16S rRNA gene clusters derived from sponge-associated microorganisms (Hentschel et al., 2002; Simister et al., 2012; Steinert et al., 2014; Taylor et al., 2007). However, many prokaryotic members of sponge-specific clusters were also found in seawater and sediment samples, albeit at low abundances, therefore these clusters should be considered as “sponge-enriched” instead of “sponge-specific” (Taylor et al., 2013; Thomas et al., 2016). Approximately half of the most abundant OTUs in the present study are significantly enriched in sponges belonging to the large collection of sponge microbiome project specimens (Moitinho-Silva et al., 2017a). These sponge-enriched OTUs belong to certain phyla and classes (e.g., *Alphaproteobacteria*, *Gammaproteobacteria*, *Deltaproteobacteria*, *Deferribacteres*, *Gemmatimonadetes*, *Nitrospirae*, *Thaumarchaeota*), which comprised many “sponge-specific” sequences in past studies (Simister et al., 2012; Taylor et al., 2007). At lower taxonomic levels, many sponge-enriched OTUs represent still uncultured prokaryotes; hence their ecological functions are still unknown (e.g., *Sva0996 marine group*, *Sh765B-TzT-29*, *PAUC34f*, *Marine Group I*). While uncultured, these taxa are often associated with sponge hosts (Moitinho-Silva et al., 2014; Steinert et al., 2017; Thomas et al., 2016; Verhoeven, Kavanagh & Dufour, 2017). In contrast, other sponge-enriched OTUs in our study belong to known nitrification and denitrification related prokaryotic taxa (e.g., *Nitrosomonadaceae*, *Desulfurellaceae*, *Nitrospinaceae*, *Nitrosococcus*, *Nitrospira*), suggesting that these symbionts may play a role in nitrogen cycling processes.

### Aerobic nitrification in Vietnamese sponges

Ammonia is known as metabolic waste products excreted by sponge cells, and the microbial nitrifiers may be key components in ammonia waste removal (Bayer, Schmitt & Hentschel, 2008; Hoffmann et al., 2009; Jiménez & Ribes, 2007). Taxonomic analysis of OTUs at lower levels showed that a large number of OTUs (*n* = 78) belonged to prokaryotic taxa whose representatives are known aerobic nitrifiers: Candidatus *Nitrosopumilus* (AOA), *Nitrosococcus*, *Nitrosomonadaceae* (AOB), and *Nitrospira* (NOB) (Fig. S6). Recent studies have demonstrated that some *Nitrospira* are also capable of performing complete ammonium oxidation (Comammox) (Daims et al., 2015; Pinto et al., 2016; Van Kessel et al., 2015). These OTUs were found in most of the Vietnamese sponges studied here (17 out of 18 species and 26 out of 27 samples), of which some species harbored a large proportion (>20% in prokaryotic communities) of these taxa (i.e., *Amphimedon* sp. 1, *T. aploos*, *C. schulzei*, *Haplosclerida* sp., *H. amboinensis*, *Niphatidae* sp.). However, the sponges investigated harbored different prokaryotic partners putatively able to perform aerobic nitrification: *Amphimedon* sp. 1, *C. schulzei*, *Haplosclerida* sp., and *Niphatidae* sp. predominantly hosted *Nitrosomonadaceae*, *T. aploos Nitrosococcus*, *H. amboinensis* Candidatus *Nitrosopumilus*, and *Dactylospongia* sp. *Nitrospira*. To date, all AOA found by these studies belonged to the phylum *Thaumarchaeota*, including the genera *Nitrosopumilus* and *Cenarchaeum*, whereas the NOB detected in sponges mostly belonged to *Nitrospira* (Cuvelier et al., 2014; Feng et al., 2016; Hoffmann et al., 2009; Kennedy et al., 2014; Kowalchuk & Stephen, 2001; Pfister, Gilbert & Gibbons, 2014; Steger et al., 2008; Turque et al., 2010; Zhang et al., 2015). In addition, *Nitrosospira* (AOB) was found in specimens *Aplysina aerophoba* from the Mediterranean Sea (Bayer, Schmitt & Hentschel, 2008). Overall, these findings suggest that nitrification is an important microbial process in marine sponges. The appearance of abundant OTUs related to prokaryotic taxa known for nitrification in Vietnamese sponges further supports this idea.

## Conclusion

In our present study we investigated the sponge-associated prokaryotic composition of 18 sponge species collected from the central coastal region of Vietnam. Our study highlights the prokaryotic diversity associated with Vietnamese sponges as well as the pattern of host-specificity among samples with replicates. The presence of significantly sponge-enriched OTUs in sponge microbiome database supports the general consensus that sponges host certain prokaryotic taxa that are not found or found only in a few samples of other environments. In addition, our study reveals the presence of prokaryotic taxa particularly known for nitrification, which indicates nitrification might be an important microbial process in sponge hosts.

## Supplemental Information

10.7717/peerj.4970/supp-1 Supplemental Information 1 Table S1. Sample data, including sample name, identified taxonomy, sampling date and site, and higher taxonomic levels of collection for each sample.

10.7717/peerj.4970/supp-2 Supplemental Information 2 Table S2. List of primers used in this study.

10.7717/peerj.4970/supp-3 Supplemental Information 3 Table S3. List of barcodes, unitag sequences and accession numbers used in this study.

10.7717/peerj.4970/supp-4 Supplemental Information 4 Table S4. Kruskal-Wallis analysis of the prokaryotic alpha diversity indices of sponge-species with duplicates. The Kruskal-Wallis test was performed using function *kruskal.test* within the FSA package in R. Significant differences are highlighted in bold.

10.7717/peerj.4970/supp-5 Supplemental Information 5 Table S5. Multivariate analysis of the influence of host-identity on their prokaryotic communities for sponge-species with replicates. Multivariate analyses were performed using the functions *betadisper*,* permutest*, and the permutational multivariate analysis of variance function (*adonis)* of the vegan package in R. Significant differences are highlighted in bold.

10.7717/peerj.4970/supp-6 Supplemental Information 6 Fig. S1. Phylogenetic tree based on 18S rRNA gene sequences (>1700 bp sequences) of sponge specimens in this study (green bold font) and their closest sequences derived from NCBI (black). The sequences were aligned using MAFFT (v.7.222) with the FFT-NS-i strategy. The phylogenetic tree was constructed using RAxML version 7.2.6 with the GTRGAMMA model and 1000 bootstrap replicates. Bootstrap value < 50 are not shown.

10.7717/peerj.4970/supp-7 Supplemental Information 7 Fig. S2. Phylogenetic tree based on COI gene sequences (>600 bp sequences) of sponge specimens in this study (green bold font) and their closest sequences derived from NCBI (black). The sequences were aligned using MAFFT (v.7.222) with the FFT-NS-i strategy. Phylogenetic tree was constructed using RAxML version 7.2.6 with the GTRGAMMA model and 1000 bootstrap replicates. Bootstrap value < 50 are not shown.

10.7717/peerj.4970/supp-8 Supplemental Information 8 Fig. S3. Rarefaction curves indicating the average observed species for the different samples using QIIME script *alpha_rarefaction.py*.

10.7717/peerj.4970/supp-9 Supplemental Information 9 Fig. S4. Heatmap and bar chart of archaeal OTUs (phylum *Thaumarchaeota*) in different sponges. OTU taxonomy was assigned by NG-tax.

10.7717/peerj.4970/supp-10 Supplemental Information 10 Fig. S5. Dendrogram displaying Bray-Curtis dissimilarity among sponge prokaryotic communities at OTU level. The hierarchical clustering based on Bray-Curtis similarity was calculated using the vegdist (method = “bray”) function of the vegan package in R version 3.3.1.

10.7717/peerj.4970/supp-11 Supplemental Information 11 Fig. S6. Heatmap and bar chart of OTUs belonging to known nitrification taxa in different sponges. OTU taxonomy was assigned by NG-tax.

## Additional Information and Declarations

### Field Study Permissions

The following information was supplied relating to field study approvals (i.e., approving body and any reference numbers): The field experiments for collecting sponge samples were approved by Vietnam Ministry of Science and Technology (2322/QĐ-BKHCN and 17/2014/HĐ-ĐTĐLCN).

### Data Availability

The following information was supplied regarding data availability: Illumina MiSeq raw sequence data can be accessed via the NCBI Sequence Read Archive with accession numbers SRS1815731–SRS1815757. The 18S rRNA and COI gene sequences can be accessed via GenBank under the accession numbers: KX894454–KX894480 (18S rRNA genes) and KX894481–KX894504 (COI genes).

## References

[ref-1] Abe T, Sahin FP, Akiyama K, Naito T, Kishigami M, Miyamoto K, Sakakibara Y, Uemura D (2012). Construction of a metagenomic library for the marine sponge *Halichondria okadai*. Bioscience, Biotechnology, and Biochemistry.

[ref-2] Alex A, Antunes A (2015). Pyrosequencing characterization of the microbiota from Atlantic intertidal marine sponges reveals high microbial diversity and the lack of co-occurrence patterns. PLOS ONE.

[ref-3] Altschul SF, Gish W, Miller W, Myers EW, Lipman DJ (1990). Basic local alignment search tool. Journal of Molecular Biology.

[ref-4] Apprill A, McNally S, Parsons R, Weber L (2015). Minor revision to V4 region SSU rRNA 806R gene primer greatly increases detection of SAR11 bacterioplankton. Aquatic Microbial Ecology.

[ref-5] Bayer K, Kamke J, Hentschel U (2014). Quantification of bacterial and archaeal symbionts in high and low microbial abundance sponges using real-time PCR. FEMS Microbiology Ecology.

[ref-6] Bayer K, Schmitt S, Hentschel U (2008). Physiology, phylogeny and in situ evidence for bacterial and archaeal nitrifiers in the marine sponge *Aplysina aerophoba*. Environmental Microbiology.

[ref-7] Bell JJ (2008). The functional roles of marine sponges. Estuarine, Coastal and Shelf Science.

[ref-8] Capone DG, Bronk DA, Mulholland MR, Carpenter EJ (2008). Nitrogen in the Marine Environment.

[ref-9] Caporaso JG, Kuczynski J, Stombaugh J, Bittinger K, Bushman FD, Costello EK, Fierer N, Peña AG, Goodrich JK, Gordon JI, Huttley GA, Kelley ST, Knights D, Koenig JE, Ley RE, Lozupone CA, McDonald D, Muegge BD, Pirrung M, Reeder J, Sevinsky JR, Turnbaugh PJ, Walters WA, Widmann J, Yatsunenko T, Zaneveld J, Knight R (2010). QIIME allows analysis of high-throughput community sequencing data. Nature Methods.

[ref-10] Cleary DFR, Becking LE, de Voogd NJ, Pires ACC, Polónia ARM, Egas C, Gomes NCM (2013). Habitat- and host-related variation in sponge bacterial symbiont communities in Indonesian waters. FEMS Microbiology Ecology.

[ref-11] Corredor JE, Wilkinson CR, Vicente VP, Morell JM, Otero E (1988). Nitrate release by Caribbean reef sponges. Limnology and Oceanography.

[ref-12] Cuc NT, Anh HLT, Hang DTT, Nhiem NX, Dang NH, Nam NH, Yen PH, Thung DC, Thuc VK, Minh CV, Kiem PV (2015). Sesquiterpenes from the Vietnamese marine sponge *Dysidea fragilis*. Natural Product Communications.

[ref-13] Cuvelier ML, Blake E, Mulheron R, McCarthy P, Blackwelder P, Vega Thurber RL, Lopez JV (2014). Two distinct microbial communities revealed in the sponge *Cinachyrella*. Frontiers in Microbiology.

[ref-14] Daims H, Lebedeva EV, Pjevac P, Han P, Herbold C, Albertsen M, Jehmlich N, Palatinszky M, Vierheilig J, Bulaev A, Kirkegaard RH, von Bergen M, Rattei T, Bendinger B, Nielsen PH, Wagner M (2015). Complete nitrification by Nitrospira bacteria. Nature.

[ref-15] Di HJ, Cameron KC, Shen J-P, Winefield CS, O’Callaghan M, Bowatte S, He J-Z (2010). Ammonia-oxidizing bacteria and archaea grow under contrasting soil nitrogen conditions. FEMS Microbiology Ecology.

[ref-16] Diaz MC, Ward BB (1997). Sponge-mediated nitrification in tropical benthic communities. Marine Ecology Progress Series.

[ref-17] Dupont S, Corre E, Li Y, Vacelet J, Bourguet-Kondracki M-L (2013). First insights into the microbiome of a carnivorous sponge. FEMS Microbiology Ecology.

[ref-18] Duris Z, Horka I, Juracka PJ, Petrusek A, Sandford F (2011). These squatters are not innocent: the evidence of parasitism in sponge-inhabiting shrimps. PLOS ONE.

[ref-19] Easson CG, Thacker RW (2014). Phylogenetic signal in the community structure of host-specific microbiomes of tropical marine sponges. Frontiers in Microbiology.

[ref-20] Edgar RC (2010). Search and clustering orders of magnitude faster than BLAST. Bioinformatics.

[ref-21] Erwin PM, Coma R, Lopez-Sendino P, Serrano E, Ribes M (2015). Stable symbionts across the HMA-LMA dichotomy: low seasonal and interannual variation in sponge-associated bacteria from taxonomically diverse hosts. FEMS Microbiology Ecology.

[ref-22] Erwin PM, Olson JB, Thacker RW (2011). Phylogenetic diversity, host-specificity and community profiling of sponge-associated bacteria in the Northern Gulf of Mexico. PLOS ONE.

[ref-23] Feng G, Sun W, Zhang F, Karthik L, Li Z (2016). Inhabitancy of active *Nitrosopumilus*-like ammonia-oxidizing archaea and *Nitrospira* nitrite-oxidizing bacteria in the sponge *Theonella swinhoei*. Scientific Reports.

[ref-24] Fisch KM, Gurgui C, Heycke N, van der Sar SA, Anderson SA, Webb VL, Taudien S, Platzer M, Rubio BK, Robinson SJ, Crews P, Piel J (2009). Polyketide assembly lines of uncultivated sponge symbionts from structure-based gene targeting. Nature Chemical Biology.

[ref-25] Geller J, Meyer C, Parker M, Hawk H (2013). Redesign of PCR primers for mitochondrial cytochrome *c* oxidase subunit I for marine invertebrates and application in all-taxa biotic surveys. Molecular Ecology Resources.

[ref-26] Giles EC, Kamke J, Moitinho-Silva L, Taylor MW, Hentschel U, Ravasi T, Schmitt S (2013). Bacterial community profiles in low microbial abundance sponges. FEMS Microbiology Ecology.

[ref-27] Gloeckner V, Wehrl M, Moitinho-Silva M, Gernert C, Schupp P, Pawlik JR, Lindquist NL, Erpenbeck D, Wörheide G, Hentschel U (2014). The HMA-LMA dichotomy revisited: an electron microscopical survey of 56 sponge species. Biological Bulletin.

[ref-28] Hardoim CCP, Costa R (2014). Microbial communities and bioactive compounds in marine sponges of the family Irciniidae—a review. Marine Drugs.

[ref-29] Hatzenpichler R (2012). Diversity, physiology, and niche differentiation of ammonia-oxidizing archaea. Applied and Environmental Microbiology.

[ref-30] Hentschel U, Hopke J, Horn M, Friedrich AB, Wagner M, Hacker J, Moore BS (2002). Molecular evidence for a uniform microbial community in sponges from different oceans. Applied and Environmental Microbiology.

[ref-31] Hentschel U, Usher KM, Taylor MW (2006). Marine sponges as microbial fermenters. FEMS Microbiology Ecology.

[ref-32] Herfort L, Schouten S, Abbas B, Veldhuis MJW, Coolen MJL, Wuchter C, Boon JP, Herndl GJ, Sinninghe Damsté JS (2007). Variations in spatial and temporal distribution of Archaea in the North Sea in relation to environmental variables. FEMS Microbiology Ecology.

[ref-33] Hoffmann F, Radax R, Woebken D, Holtappels M, Lavik G, Rapp HT, Schlappy ML, Schleper C, Kuypers MM (2009). Complex nitrogen cycling in the sponge *Geodia barretti*. Environmental Microbiology.

[ref-34] Hong J-K, Cho J-C (2015). Environmental variables shaping the ecological niche of thaumarchaeota in soil: direct and indirect causal effects. PLOS ONE.

[ref-35] Indraningrat AAG, Smidt H, Sipkema D (2016). Bioprospecting sponge-associated microbes for antimicrobial compounds. Marine Drugs.

[ref-36] Isobe K, Koba K, Suwa Y, Ikutani J, Fang Y, Yoh M, Mo J, Otsuka S, Senoo K (2012). High abundance of ammonia-oxidizing archaea in acidified subtropical forest soils in southern China after long-term N deposition. FEMS Microbiology Ecology.

[ref-37] Jackson SA, Flemer B, McCann A, Kennedy J, Morrissey JP, O’Gara F, Dobson ADW (2013). Archaea appear to dominate the microbiome of *Inflatella pellicula* deep sea sponges. PLOS ONE.

[ref-38] Jasmin C, Anas A, Nair S (2015). Bacterial diversity associated with *Cinachyra cavernosa* and *Haliclona pigmentifera*, cohabiting sponges in the coral reef ecosystem of Gulf of Mannar, Southeast Coast of India. PLOS ONE.

[ref-39] Jiménez E, Ribes M (2007). Sponges as a source of dissolved inorganic nitrogen: nitrification mediated by temperate sponges. Limnology and Oceanography.

[ref-40] Katoh K, Standley DM (2013). MAFFT multiple sequence alignment software version 7: improvements in performance and usability. Molecular Biology and Evolution.

[ref-41] Kearse M, Moir R, Wilson A, Stones-Havas S, Cheung M, Sturrock S, Buxton S, Cooper A, Markowitz S, Duran C, Thierer T, Ashton B, Meintjes P, Drummond A (2012). Geneious basic: an integrated and extendable desktop software platform for the organization and analysis of sequence data. Bioinformatics.

[ref-42] Kennedy J, Flemer B, Jackson SA, Morrissey JP, O’Gara F, Dobson ADW (2014). Evidence of a putative deep sea specific microbiome in marine sponges. PLOS ONE.

[ref-43] Khan ST, Musarrat J, Alkhedhairy AA, Kazuo S (2013). Diversity of bacteria and polyketide synthase associated with marine sponge *Haliclona* sp. Annals of Microbiology.

[ref-44] Kiem PV, Minh CV, Nhiem NX, Cuc NT, Quang NV, Tuan Anh HL, Tai BH, Yen PH, Hoai NT, Ho KY, Kim N, Park S, Kim SH (2014). Muurolane-type sesquiterpenes from marine sponge *Dysidea cinerea*. Magnetic Resonance in Chemistry.

[ref-45] Kiem PV, Nhiem NX, Tai BH, Anh HLT, Hang DTT, Cuc NT, Huyen LT, Nam NH, Yen PH, Thung DC, Minh CV (2016). Bis-sesquiterpene from the marine sponge *Dysidea fragilis*. Natural Product Communications.

[ref-46] Kirchman DL, Elifantz H, Dittel AI, Malmstrom RR, Cottrell MT (2007). Standing stocks and activity of Archaea and bacteria in the western Arctic Ocean. Limnology and Oceanography.

[ref-47] Kowalchuk GA, Stephen JR (2001). Ammonia-oxidizing bacteria: a model for molecular microbial ecology. Annual Review of Microbiology.

[ref-48] Lattig P, Martín D (2011). Sponge-associated Haplosyllis (Polychaeta: Syllidae: Syllinae) from the Caribbean Sea, with the description of four new species. Scientia Marina.

[ref-49] Lee OO, Wang Y, Yang J, Lafi FF, Al-Suwailem A, Qian P-Y (2011). Pyrosequencing reveals highly diverse and species-specific microbial communities in sponges from the Red Sea. ISME Journal.

[ref-50] Li Z, Hu Y, Liu Y, Huang Y, He L, Miao X (2007). 16S rDNA clone library-based bacterial phylogenetic diversity associated with three South China Sea sponges. World Journal of Microbiology and Biotechnology.

[ref-51] Li CQ, Liu WC, Zhu P, Yang JL, Cheng KD (2011). Phylogenetic diversity of bacteria associated with the marine sponge *Gelliodes carnosa* collected from the Hainan Island coastal waters of the South China Sea. Microbial Ecology.

[ref-52] Linh VTT, Kiem DT, Ngoc PH, Phu LH, Tam PH, Vinh LT (2015). Coastal sea water quality of Nha Trang bay, Khanh Hoa, Viet Nam. Journal of Shipping and Ocean Engineering.

[ref-53] Maldonado M, Ribes M, van Duyl FC (2012). Nutrient fluxes through sponges: biology, budgets, and ecological implications. Advances in Marine Biology.

[ref-54] Maloof AC, Rose CV, Beach R, Samuels BM, Calmet CC, Erwin DH, Poirier GR, Yao N, Simons FJ (2010). Possible animal-body fossils in pre-Marinoan limestones from South Australia. Nature Geoscience.

[ref-55] Margot H, Acebal C, Toril E, Amils R, Fernandez Puentes J (2002). Consistent association of crenarchaeal Archaea with sponges of the genus Axinella. Marine Biology.

[ref-56] Medlin L, Elwood HJ, Stickel S, Sogin ML (1988). The characterization of enzymatically amplified eukaryotic 16S-like rRNA-coding regions. Gene.

[ref-57] Moitinho-Silva L, Bayer K, Cannistraci CV, Giles EC, Ryu T, Seridi L, Ravasi T, Hentschel U (2014). Specificity and transcriptional activity of microbiota associated with low and high microbial abundance sponges from the Red Sea. Molecular Ecology.

[ref-58] Moitinho-Silva L, Nielsen S, Amir A, Gonzalez A, Ackermann GL, Cerrano C, Astudillo-Garcia C, Easson C, Sipkema D, Liu F, Steinert G, Kotoulas G, McCormack GP, Feng G, Bell JJ, Vicente J, Björk JR, Montoya JM, Olson JB, Reveillaud J, Steindler L, Pineda M-C, Marra MV, Ilan M, Taylor MW, Polymenakou P, Erwin PM, Schupp PJ, Simister RL, Knight R, Thacker RW, Costa R, Hill RT, Lopez-Legentil S, Dailianis T, Ravasi T, Hentschel U, Li Z, Webster NS, Thomas T (2017a). The sponge microbiome project. GigaScience.

[ref-59] Moitinho-Silva L, Steinert G, Nielsen S, Hardoim CCP, Wu Y-C, McCormack GP, López-Legentil S, Marchant R, Webster N, Thomas T, Hentschel U (2017b). Predicting the HMA-LMA status in marine sponges by machine learning. Frontiers in Microbiology.

[ref-60] Ministry of Natural Resources and Environment (MONRE) (2016). National Environmental Status in the Period from 2011 to 2015.

[ref-61] Naim MA, Morillo JA, Sorensen SJ, Waleed AA, Smidt H, Sipkema D (2014). Host-specific microbial communities in three sympatric North Sea sponges. FEMS Microbiology Ecology.

[ref-62] Nguyen HT, Chau VM, Tran TH, Phan VK, Hoang TH, Nguyen TD, Nguyen XN, Tai BH, Hyun JH, Kang HK, Kim YH (2009). C29 sterols with a cyclopropane ring at C-25 and 26 from the Vietnamese marine sponge *Ianthella* sp. and their anticancer properties. Bioorganic & Medicinal Chemistry Letters.

[ref-63] Nguyen XC, Longeon A, Pham VC, Urvois F, Bressy C, Trinh TT, Nguyen HN, Phan VK, Chau VM, Briand JF, Bourguet-Kondracki ML (2013). Antifouling 26,27-cyclosterols from the Vietnamese marine sponge *Xestospongia testudinaria*. Journal of Natural Products.

[ref-64] Nguyen XN, Nguyen TC, Dan TT, Do TT, Nguyen HN, Pham HY, Do CT, Vu KT, Hoang le TA, Bui HT, Chau VM, Phan VK (2015). ^1^H and ^13^C NMR assignments of sesquiterpenes from *Dysidea fragilis*. Magnetic Resonance in Chemistry.

[ref-65] Ogle DH (2017). FSA: Fisheries Stock Analysis.

[ref-66] Oksanen J, Blanchett FG, Kindt R, Legendre P, Minchin PR, O’Hara RB, Simpson GL, Solymos P, Stevens MHM, Wagner H (2016). https://github.com/vegandevs/vegan.

[ref-67] Oton EV, Quince C, Nicol GW, Prosser JI, Gubry-Rangin C (2015). Phylogenetic congruence and ecological coherence in terrestrial Thaumarchaeota. ISME Journal.

[ref-68] Pfister CA, Gilbert JA, Gibbons SM (2014). The role of macrobiota in structuring microbial communities along rocky shores. PeerJ.

[ref-69] Pinto AJ, Marcus DN, Ijaz UZ, Bautista-de Lose Santos QM, Dick GJ, Raskin L (2016). Metagenomic evidence for the presence of Comammox Nitrospira-like bacteria in a drinking water system. mSphere.

[ref-70] Pita L, Turon X, López-Legentil S, Erwin PM (2013). Host rules: spatial stability of bacterial communities associated with marine sponges (*Ircinia* spp.) in the Western Mediterranean Sea. FEMS Microbiology Ecology.

[ref-71] Polónia ARM, Cleary DFR, Duarte LN, de Voogd NJ, Gomes NCM (2014). Composition of Archaea in seawater, sediment, and sponges in the Kepulauan Seribu reef system, Indonesia. Microbial Ecology.

[ref-72] Prosser JI, Nicol GW (2008). Relative contributions of archaea and bacteria to aerobic ammonia oxidation in the environment. Environmental Microbiology.

[ref-73] Quang Tri Center for Environmental Engineering and Monitoring (QCEEM) (2015). Monitoring Report of Environmental Quality of Quang Tri Province in 2015.

[ref-74] Quang TM (2013). A review of the diversity of sponges (Porifera) in Vietnam.

[ref-75] Radax R, Hoffmann F, Rapp HT, Leininger S, Schleper C (2012). Ammonia-oxidizing archaea as main drivers of nitrification in cold-water sponges. Environmental Microbiology.

[ref-76] Ramiro-Garcia J, Hermes GDA, Giatsis C, Sipkema D, Zoetendal EG, Schaap PJ, Smidt H (2016). NG-tax, a highly accurate and validated pipeline for analysis of 16S rRNA amplicons from complex biomes. F1000Research.

[ref-77] R Core Team (2016). R: A Language and Environment for Statistical Computing.

[ref-78] Rodríguez-Marconi S, De la Iglesia R, Díez B, Fonseca CA, Hajdu E, Trefault N (2015). Characterization of bacterial, Archaeal and Eukaryote symbionts from Antarctic sponges reveals a high diversity at a three-domain level and a particular signature for this ecosystem. PLOS ONE.

[ref-79] Santavy DL, Harmelin Vivien M, Salvat B (1985). The symbiotic relationship between a blue-pigmented bacterium and the coral reef sponge, *Terpios granulosa*. Proceedings of the Fifth International Coral Reef Congress, Tahiti.

[ref-80] Santavy DL, Colwell RR (1990). Comparison of bacterial communities associated with the Caribbean selerosponge *Ceratoporella nicholsoni* and ambient seawater. Marine Ecology Progress Series.

[ref-81] Sarà M (1971). Ultrastructural aspects of the symbiosis between two species of the genus *Aphanocapsa* (Cyanophyceae) and *Ircinia variabilis* (Demospongiae). Marine Biology.

[ref-82] Schmitt S, Deines P, Behnam F, Wagner M, Taylor MW (2011). Chloroflexi bacteria are more diverse, abundant, and similar in high than in low microbial abundance sponges. FEMS Microbiology Ecology.

[ref-83] Simion P, Philippe H, Baurain D, Jager M, Richter DJ, Di Franco A, Roure B, Satoh N, Quéinnec É, Ereskovsky A, Lapébie P, Corre E, Delsuc F, King N, Wörheide G, Manuel M (2017). A large and consistent phylogenomic dataset supports sponges as the sister group to all other animals. Current Biology.

[ref-84] Simister RL, Deines P, Botté ES, Webster NS, Taylor MW (2012). Sponge-specific clusters revisited: a comprehensive phylogeny of sponge-associated microorganisms. Environmental Microbiology.

[ref-85] Sipkema D, Holmes B, Nichols SA, Blanch HW (2009). Biological characterisation of *Haliclona* (*?gelliu*s) sp.: sponge and associated microorganisms. Microbial Ecology.

[ref-86] Stamatakis A (2014). RAxML version 8: a tool for phylogenetic analysis and post-analysis of large phylogenies. Bioinformatics.

[ref-87] Steger D, Ettinger-Epstein P, Whalan S, Hentschel U, De Nys R, Wagner M, Taylor MW (2008). Diversity and mode of transmission of ammonia-oxidizing archaea in marine sponges. Environmental Microbiology.

[ref-88] Steinert G, Rohde S, Janussen D, Blaurock C, Schupp PJ (2017). Host-specific assembly of sponge-associated prokaryotes at high taxonomic ranks. Scientific Reports.

[ref-89] Steinert G, Taylor MW, Deines P, Simister RL, de Voogd NJ, Hoggard M, Schupp PJ (2016). In four shallow and mesophotic tropical reef sponges from Guam the microbial community largely depends on host identity. PeerJ.

[ref-90] Steinert G, Whitfield S, Taylor MW, Thoms C, Schupp PJ (2014). Application of diffusion growth chambers for the cultivation of marine sponge-associated bacteria. Marine Biotechnology.

[ref-91] Tang SL, Hong MJ, Liao MH, Jane WN, Chiang PW, Chen CB, Chen CA (2011). Bacteria associated with an encrusting sponge (*Terpios hoshinota*) and the corals partially covered by the sponge. Environmental Microbiology.

[ref-92] Taylor MW, Radax R, Steger D, Wagner M (2007). Sponge-associated microorganisms: evolution, ecology, and biotechnological potential. Microbiology and Molecular Biology Reviews.

[ref-93] Taylor MW, Tsai P, Simister RL, Deines P, Botte E, Ericson G, Schmitt S, Webster NS (2013). ‘Sponge-specific’ bacteria are widespread (but rare) in diverse marine environments. ISME Journal.

[ref-94] Thomas T, Moitinho-Silva L, Lurgi M, Bjork JR, Easson C, Astudillo-Garcia C, Olson JB, Erwin PM, Lopez-Legentil S, Luter H, Chaves-Fonnegra A, Costa R, Schupp PJ, Steindler L, Erpenbeck D, Gilbert J, Knight R, Ackermann G, Victor Lopez J, Taylor MW, Thacker RW, Montoya JM, Hentschel U, Webster NS (2016). Diversity, structure and convergent evolution of the global sponge microbiome. Nature Communications.

[ref-95] Tolar B, King G, Hollibaugh J (2013). An analysis of Thaumarchaeota populations from the Northern Gulf of Mexico. Frontiers in Microbiology.

[ref-96] Turque AS, Batista D, Silveira CB, Cardoso AM, Vieira RP, Moraes FC, Clementino MM, Albano RM, Paranhos R, Martins OB, Muricy G (2010). Environmental shaping of sponge associated archaeal communities. PLOS ONE.

[ref-97] Unson MD, Faulkner DJ (1993). Cyanobacterial symbiont biosynthesis of chlorinated metabolites from *Dysidea herbacea* (Porifera). Experientia.

[ref-98] Vacelet J, Donadey C (1977). Electron microscope study of the association between some sponges and bacteria. Journal of Experimental Marine Biology and Ecology.

[ref-99] Van Kessel MAHJ, Speth DR, Albertsen M, Nielsen PH, Op den Camp HJM, Kartal B, Jetten MSM, Lücker S (2015). Complete nitrification by a single microorganism. Nature.

[ref-100] Van Soest RWM, Boury-Esnault N, Vacelet J, Dohrmann M, Erpenbeck D, De Voogd NJ, Santodomingo N, Vanhoorne B, Kelly M, Hooper JNA (2012). Global diversity of sponges (Porifera). PLOS ONE.

[ref-101] Verhamme DT, Prosser JI, Nicol GW (2011). Ammonia concentration determines differential growth of ammonia-oxidising archaea and bacteria in soil microcosms. ISME Journal.

[ref-102] Verhoeven JTP, Kavanagh AN, Dufour SC (2017). Microbiome analysis shows enrichment for specific bacteria in separate anatomical regions of the deep-sea carnivorous sponge *Chondrocladia grandis*. FEMS Microbiology Ecology.

[ref-103] Ward BB, Ward BB, Arp DJ, Klotz MG (2011). Nitrification in the ocean. Nitrification.

[ref-104] Warnes GR, Bolker B, Bonebakker L, Gentleman R, Liaw WHA, Lumley T, Maechler M, Magnusson A, Moeller S, Schwartz M, Venables B (2016). https://CRAN.R-project.org/package=gplots.

[ref-105] Webster SN, Watts EMJ, Hill TR (2001). Detection and phylogenetic analysis of novel Crenarchaeote and Euryarchaeote 16S ribosomal RNA gene sequences from a Great Barrier Reef Sponge. Marine Biotechnology.

[ref-106] Weigel BL, Erwin PM (2015). Intraspecific variation in microbial symbiont communities of the Sun Sponge, *Hymeniacidon heliophila*, from intertidal and subtidal habitats. Applied and Environmental Microbiology.

[ref-107] Wilkinson CR (1978a). Microbial associations in sponges. III. Ultrastructure of the in situ associations in coral reef sponges. Marine Biology.

[ref-108] Wilkinson CR (1978b). Significance of microbial symbionts in sponge evolution and ecology. Symbiosis.

[ref-109] Wuchter C, Abbas B, Coolen MJL, Herfort L, van Bleijswijk J, Timmers P, Strous M, Teira E, Herndl GJ, Middelburg JJ, Schouten S, Sinninghe Damsté JS (2006). Archaeal nitrification in the ocean. Proceedings of the National Academy of Sciences of the United States of America.

[ref-110] Yao H, Campbell CD, Chapman SJ, Freitag TE, Nicol GW, Singh BK (2013). Multi-factorial drivers of ammonia oxidizer communities: evidence from a national soil survey. Environmental Microbiology.

[ref-111] Yilmaz P, Parfrey LW, Yarza P, Gerken J, Pruesse E, Quast C, Schweer T, Peplies J, Ludwig W, Glockner FO (2014). The SILVA and “all-species living tree project (LTP)” taxonomic frameworks. Nucleic Acids Research.

[ref-112] Yin Z, Zhu M, Davidson EH, Bottjer DJ, Zhao F, Tafforeau P (2015). Sponge grade body fossil with cellular resolution dating 60 Myr before the Cambrian. Proceedings of the National Academy of Sciences of the United States of America.

[ref-113] Zhang F, Pita L, Erwin PM, Abaid S, López-Legentil S, Hill RT (2015). Symbiotic archaea in marine sponges show stability and host specificity in community structure and ammonia oxidation functionality. FEMS Microbiology Ecology.

