# Supplementary material for: Archaeal and bacterial diversity and community composition from 18 phylogenetically divergent sponge species in Vietnam
Source: PeerJ. 2018 Jun 8;6:e4970. doi: 10.7717/peerj.4970 (PMC5995103; doi:10.7717/peerj.4970)
Supplement: Supplemental Information 1 [file peerj-06-4970-s001.docx]

| **Sample** | **Taxonomy** | **Date** | **Site** | **Coordinates** | **Class** | **Order** | **Family** |
| --- | --- | --- | --- | --- | --- | --- | --- |
| AMC | *Amphimedon* sp. 1. | May-2015 | Lang Co Bay | 108°07'10.5"E; 16°13'31.6"N | Demospongiae | Haplosclerida | *Niphatidae* |
| AMQ | *Amphimedon* sp. 2 | Sep-2015 | Hon Mun Island | 109°18'08.1"E; 12°10'04.4"N | Demospongiae | Haplosclerida | *Niphatidae* |
| AXT.1 | *Axinyssa* sp. | Sep-2015 | Hon Mun Island | 109°18'08.1"E; 12°10'04.4"N | Demospongiae | Suberitida | *Halichondriidae* |
| AXT.2 | *Axinyssa* sp. | Sep-2015 | Hon Mun Island | 109°18'08.1"E; 12°10'04.4"N | Demospongiae | Suberitida | *Halichondriidae* |
| AXT.3 | *Axinyssa* sp. | Sep-2015 | Hon Mun Island | 109°18'08.1"E; 12°10'04.4"N | Demospongiae | Suberitida | *Halichondriidae* |
| AXT.4 | *Axinyssa* sp. | May-2015 | Lang Co Bay | 108°07'10.5"E; 16°13'31.6"N | Demospongiae | Suberitida | *Halichondriidae* |
| AXC | *Axos cliftoni* | Aug-2015 | Con Co Island | 107°07'06.0"E; 17°04'50.2"N | Demospongiae | Tethyida | *Hemiasterellidae* |
| CIS | *Cinachyrella schulzei* | May-2015 | Lang Co Bay | 108°07'10.5"E; 16°13'31.6"N | Demospongiae | Spirophorida | *Tetillidae* |
| CLR.1 | *Clathria reinwardti* | Sep-2015 | Hon Mun Island | 109°18'08.1"E; 12°10'04.4"N | Demospongiae | Poecilosclerida | *Microcionidae* |
| CLR.2 | *Clathria reinwardti* | Aug-2015 | Con Co Island | 107°07'06.0"E; 17°04'50.2"N | Demospongiae | Poecilosclerida | *Microcionidae* |
| DAS.1 | *Dactylospongia* sp. | May-2015 | Lang Co Bay | 108°07'10.5"E; 16°13'31.6"N | Demospongiae | Dictyoceratida | *Thorectidae* |
| DAS.2 | *Dactylospongia* sp. | Sep-2015 | Hon Mun Island | 109°18'08.1"E; 12°10'04.4"N | Demospongiae | Dictyoceratida | *Thorectidae* |
| HAS | *Halichondria* sp. | Aug-2015 | Con Co Island | 107°07'06.0"E; 17°04'50.2"N | Demospongiae | Suberitida | *Halichondriidae* |
| HAA.1 | *Haliclona amboinensis* | May-2015 | Lang Co Bay | 108°07'10.5"E; 16°13'31.6"N | Demospongiae | Haplosclerida | *Chalinidae* |
| HAA.2 | *Haliclona amboinensis* | May-2015 | Lang Co Bay | 108°07'10.5"E; 16°13'31.6"N | Demospongiae | Haplosclerida | *Chalinidae* |
| HAF | *Haliclona fascigera* | May-2015 | Lang Co Bay | 108°07'10.5"E; 16°13'31.6"N | Demospongiae | Haplosclerida | *Chalinidae* |
| CRV | *Haplosclerida* sp. | Sep-2015 | Hon Mun Island | 109°18'08.1"E; 12°10'04.4"N | Demospongiae | Haplosclerida | *-* |
| NIS | *Niphatidae* sp. | Sep-2015 | Hon Mun Island | 109°18'08.1"E; 12°10'04.4"N | Demospongiae | Haplosclerida | *Niphatidae* |
| RHG | *Rhabdastrella globostellata* | May-2015 | Lang Co Bay | 108°07'10.5"E; 16°13'31.6"N | Demospongiae | Tetractinellida | *Ancorinidae* |
| SPV | *Spheciospongia* sp. | Aug-2015 | Con Co Island | 107°07'06.0"E; 17°04'50.2"N | Demospongiae | Clionaida | *Clionaidae* |
| SPS.1 | *Spirastrella* sp. | Sep-2015 | Hon Mun Island | 109°18'08.1"E; 12°10'04.4"N | Demospongiae | Clionaida | *Spirastrellidae* |
| SPS.2 | *Spirastrella* sp. | Aug-2015 | Con Co Island | 107°07'06.0"E; 17°04'50.2"N | Demospongiae | Clionaida | *Spirastrellidae* |
| TES | *Tedania* sp. | Sep-2015 | Hon Mun Island | 109°18'08.1"E; 12°10'04.4"N | Demospongiae | Poecilosclerida | *Tedaniidae* |
| TEA | *Terpios aploos* | May-2015 | Lang Co Bay | 108°07'10.5"E; 16°13'31.6"N | Demospongiae | Hadromerida | *Suberitidae* |
| XES.1 | *Xestospongia testudinaria* | May-2015 | Lang Co Bay | 108°07'10.5"E; 16°13'31.6"N | Demospongiae | Haplosclerida | *Petrosiidae* |
| XES.2 | *Xestospongia testudinaria* | May-2015 | Lang Co Bay | 108°07'10.5"E; 16°13'31.6"N | Demospongiae | Haplosclerida | *Petrosiidae* |
| XES.3 | *Xestospongia testudinaria* | Aug-2015 | Con Co Island | 107°07'06.0"E; 17°04'50.2"N | Demospongiae | Haplosclerida | *Petrosiidae* |
