# Supplementary material for: Archaeal and bacterial diversity and community composition from 18 phylogenetically divergent sponge species in Vietnam
Source: PeerJ. 2018 Jun 8;6:e4970. doi: 10.7717/peerj.4970 (PMC5995103; doi:10.7717/peerj.4970)
Supplement: Supplemental Information 2 [file peerj-06-4970-s002.docx]

| **Primer** | **Sequence (5'-3')** | **Target** | **Annealing temperature** | **Amplicon length (bp)** | **Use** | **Reference** |
| --- | --- | --- | --- | --- | --- | --- |
| EUKF | aacctggttgatcctgccagt | Eukaryotes 18S rRNA gene | 55 | 1700-1800 | Clone library | Medlin et al., 1998 |
| EUKR | tgatccttctgcaggttcacctac | Eukaryotes 18S rRNA gene | 55 | 1700-1800 | Clone library | Medlin et al., 1998 |
| jgLCO1490 | titciaciaaycayaargayattgg | Mitochondrial COI  gene | 52 | 640 | Clone library | Geller et al., 2013 |
| jgHCO2198 | taiiacytciggrtgiccraaraayca | Mitochondrial COI gene | 52 | 640 | Clone library | Geller et al., 2013 |
| 515F | gtgycagcmgccgcggtaa | V4 region of 16S rRNA gene | 56 | 291 | Illumina sequencing | Apprill et al., 2015 |
| 806R | ggactacnvgggtwtctaat | V4 region of 16S rRNA gene | 56 | 291 | Illumina sequencing | Apprill et al., 2015 |
