# Supplementary material for: Archaeal and bacterial diversity and community composition from 18 phylogenetically divergent sponge species in Vietnam
Source: PeerJ. 2018 Jun 8;6:e4970. doi: 10.7717/peerj.4970 (PMC5995103; doi:10.7717/peerj.4970)
Supplement: Supplemental Information 3 [file peerj-06-4970-s003.docx]

| **Sample** | **Barcode** | **UniTag1** | **UniTag2** | **Accession Number** |
| --- | --- | --- | --- | --- |
| AXT.1 | CCGTCTGC | GAGCCGTAGCCAGTCTGC | GCCGTGACCGTGACATCG | SRS1815746 |
| AXT.2 | TAATACGT | GAGCCGTAGCCAGTCTGC | GCCGTGACCGTGACATCG | SRS1815747 |
| AXT.3 | GGCCAGTA | GAGCCGTAGCCAGTCTGC | GCCGTGACCGTGACATCG | SRS1815737 |
| AXT.4 | CTGAGTTC | GAGCCGTAGCCAGTCTGC | GCCGTGACCGTGACATCG | SRS1815749 |
| XES.1 | TTCTGAAC | GAGCCGTAGCCAGTCTGC | GCCGTGACCGTGACATCG | SRS1815757 |
| XES.2 | CTTCATGG | GAGCCGTAGCCAGTCTGC | GCCGTGACCGTGACATCG | SRS1815750 |
| XES.3 | CGCGCCAG | GAGCCGTAGCCAGTCTGC | GCCGTGACCGTGACATCG | SRS1815738 |
| CLR.1 | GCCTCATC | GAGCCGTAGCCAGTCTGC | GCCGTGACCGTGACATCG | SRS1815736 |
| CLR.2 | TCAGGCGA | GAGCCGTAGCCAGTCTGC | GCCGTGACCGTGACATCG | SRS1815744 |
| SPS.1 | CGTCTGAG | GAGCCGTAGCCAGTCTGC | GCCGTGACCGTGACATCG | SRS1815745 |
| SPS.2 | CGTCCTCC | GAGCCGTAGCCAGTCTGC | GCCGTGACCGTGACATCG | SRS1815740 |
| DAS.1 | ATGTTCCA | GAGCCGTAGCCAGTCTGC | GCCGTGACCGTGACATCG | SRS1815741 |
| DAS.2 | GGTCAGAT | GAGCCGTAGCCAGTCTGC | GCCGTGACCGTGACATCG | SRS1815751 |
| HAA.1 | GGAGTATG | GAGCCGTAGCCAGTCTGC | GCCGTGACCGTGACATCG | SRS1815734 |
| HAA.2 | TACTTATC | GAGCCGTAGCCAGTCTGC | GCCGTGACCGTGACATCG | SRS1815733 |
| AMC | ATCTCAGT | GAGCCGTAGCCAGTCTGC | GCCGTGACCGTGACATCG | SRS1815743 |
| CIS | TGGTTGAC | GAGCCGTAGCCAGTCTGC | GCCGTGACCGTGACATCG | SRS1815732 |
| NIS | ATAAGGTC | GAGCCGTAGCCAGTCTGC | GCCGTGACCGTGACATCG | SRS1815753 |
| HAF | GGAGCGCA | GAGCCGTAGCCAGTCTGC | GCCGTGACCGTGACATCG | SRS1815735 |
| AMQ | GTCAACGT | GAGCCGTAGCCAGTCTGC | GCCGTGACCGTGACATCG | SRS1815731 |
| CRV | CGACCGCG | GAGCCGTAGCCAGTCTGC | GCCGTGACCGTGACATCG | SRS1815739 |
| RHG | CCAAGTCA | GAGCCGTAGCCAGTCTGC | GCCGTGACCGTGACATCG | SRS1815754 |
| SPV | GAGTTCAT | GAGCCGTAGCCAGTCTGC | GCCGTGACCGTGACATCG | SRS1815742 |
| HAS | AATAAGGA | GAGCCGTAGCCAGTCTGC | GCCGTGACCGTGACATCG | SRS1815748 |
| TES | TCGTCGCC | GAGCCGTAGCCAGTCTGC | GCCGTGACCGTGACATCG | SRS1815752 |
| TEA | CTGGATAA | GAGCCGTAGCCAGTCTGC | GCCGTGACCGTGACATCG | SRS1815756 |
| AXC | AGAGATAA | GAGCCGTAGCCAGTCTGC | GCCGTGACCGTGACATCG | SRS1815755 |
