# Supplementary material for: Archaeal and bacterial diversity and community composition from 18 phylogenetically divergent sponge species in Vietnam
Source: PeerJ. 2018 Jun 8;6:e4970. doi: 10.7717/peerj.4970 (PMC5995103; doi:10.7717/peerj.4970)
Supplement: Supplemental Information 4 — The Kruskal-Wallis test was performed using function kruskal.test within the FSA package in R. Significant differences are highlighted in bold. [file peerj-06-4970-s004.docx]

| **Index** | **df** | ***Kruskal-Wallis chi-squared*** | ***p-value*** |
| --- | --- | --- | --- |
| Richness | 5 | 11.837 | **0.03709** |
| Shannon | 5 | 11.967 | **0.03525** |
| Evenness | 5 | 13.087 | **0.02257** |
| Inverse Simpson | 5 | 12.742 | **0.02592** |
