# Supplementary material for: Archaeal and bacterial diversity and community composition from 18 phylogenetically divergent sponge species in Vietnam
Source: PeerJ. 2018 Jun 8;6:e4970. doi: 10.7717/peerj.4970 (PMC5995103; doi:10.7717/peerj.4970)
Supplement: Supplemental Information 5 — Multivariate analyses were performed using the functions betadisper, permutest, and the permutational multivariate analysis of variance function (adonis) of the vegan package in R. Significant differences are highlighted in bold. [file peerj-06-4970-s005.docx]

|  | **Betadisper** | | | | |
| --- | --- | --- | --- | --- | --- |
|  | **df** | ***Sum Sq*** | ***Mean Sq*** | ***F-value*** | ***p-value*** |
| Sponge-species group | 5 | 0.044123 | 0.008825 | 2.4873 | 0.1019 |
| Residuals | 9 | 0.031931 | 0.003548 |  |  |
| Total | 14 | 0.076054 |  |  |  |
|  | **Adonis** | | | | |
|  | **df** | ***Sum Sq*** | ***Mean Sq*** | ***R^2^*** | ***p-value*** |
| Sponge-species group | 5 | 4.6035 | 0.92069 | 0.94 | **0.000999** |
| Residuals | 9 | 0.2936 | 0.03263 | 0.06 |  |
| Total | 14 | 4.8971 |  | 1 |  |
