# Supplementary figures and images for: Archaeal and bacterial diversity and community composition from 18 phylogenetically divergent sponge species in Vietnam
Source: PeerJ. 2018 Jun 8;6:e4970. doi: 10.7717/peerj.4970 (PMC5995103; doi:10.7717/peerj.4970)

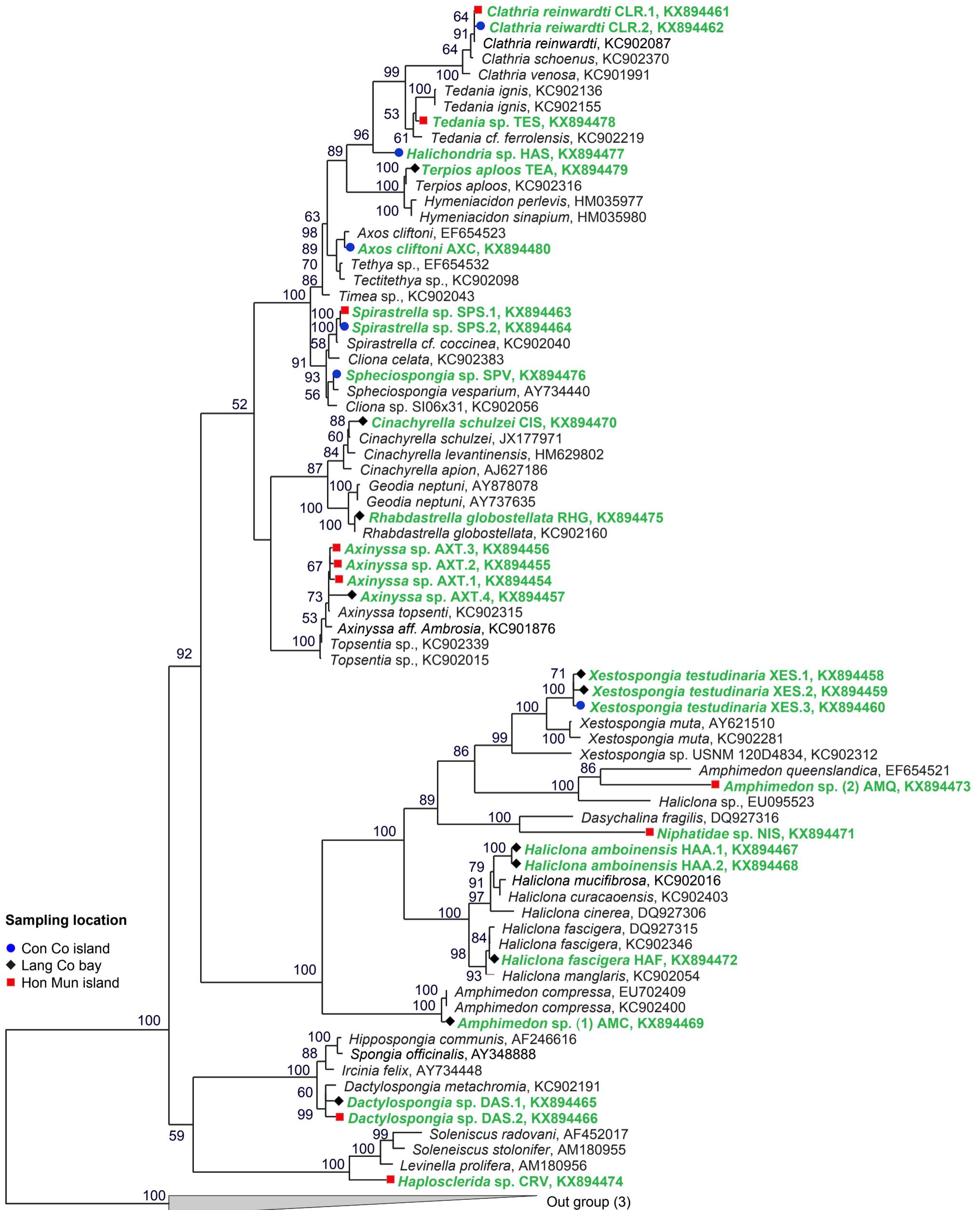

Supplement: Supplemental Information 6 — The sequences were aligned using MAFFT (v.7.222) with the FFT-NS-i strategy. The phylogenetic tree was constructed using RAxML version 7.2.6 with the GTRGAMMA model and 1000 bootstrap replicates. Bootstrap value < 50 are not shown. [file peerj-06-4970-s006.pdf]

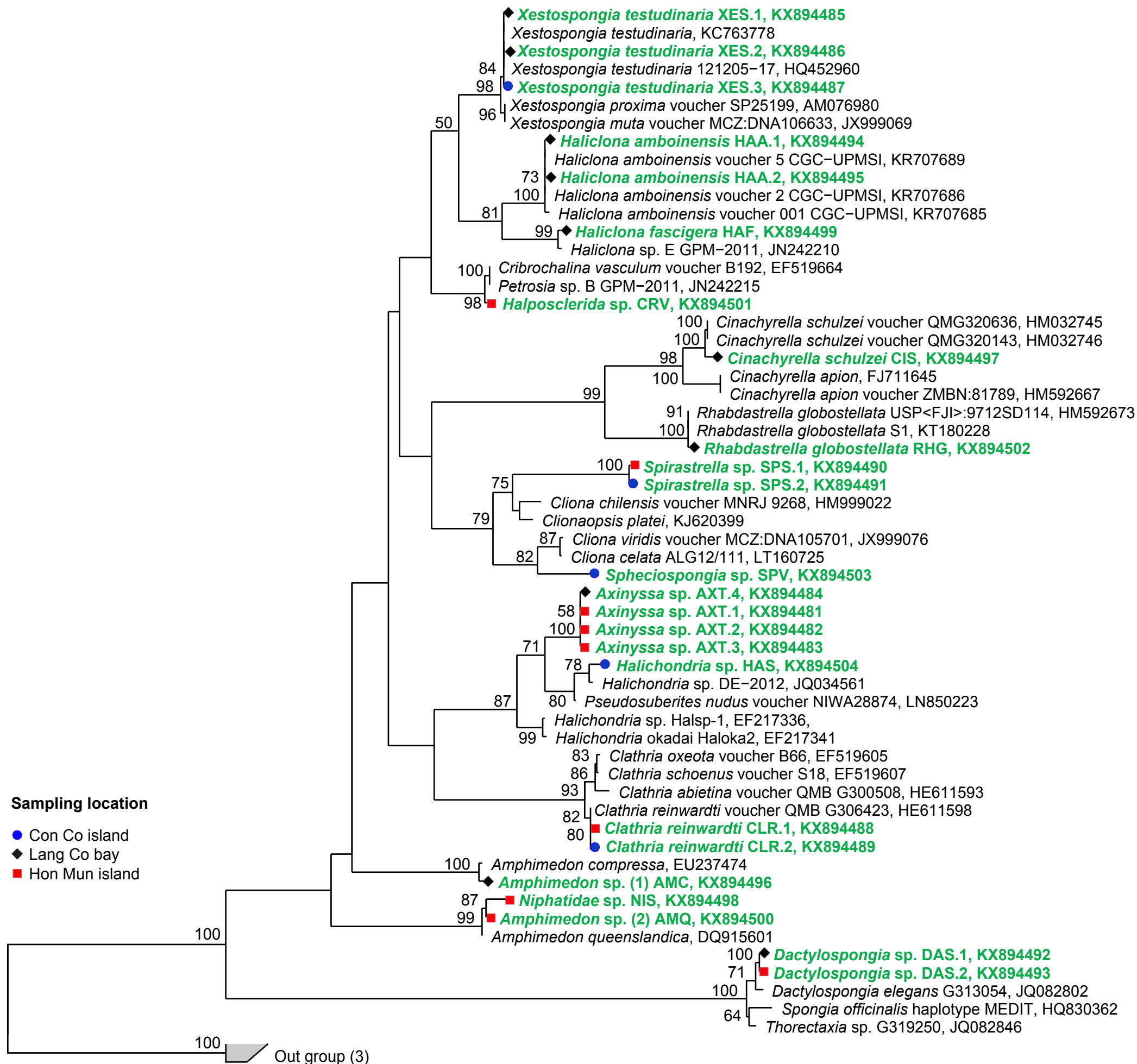

0.10

Supplement: Supplemental Information 7 — The sequences were aligned using MAFFT (v.7.222) with the FFT-NS-i strategy. Phylogenetic tree was constructed using RAxML version 7.2.6 with the GTRGAMMA model and 1000 bootstrap replicates. Bootstrap value < 50 are not shown. [file peerj-06-4970-s007.pdf]

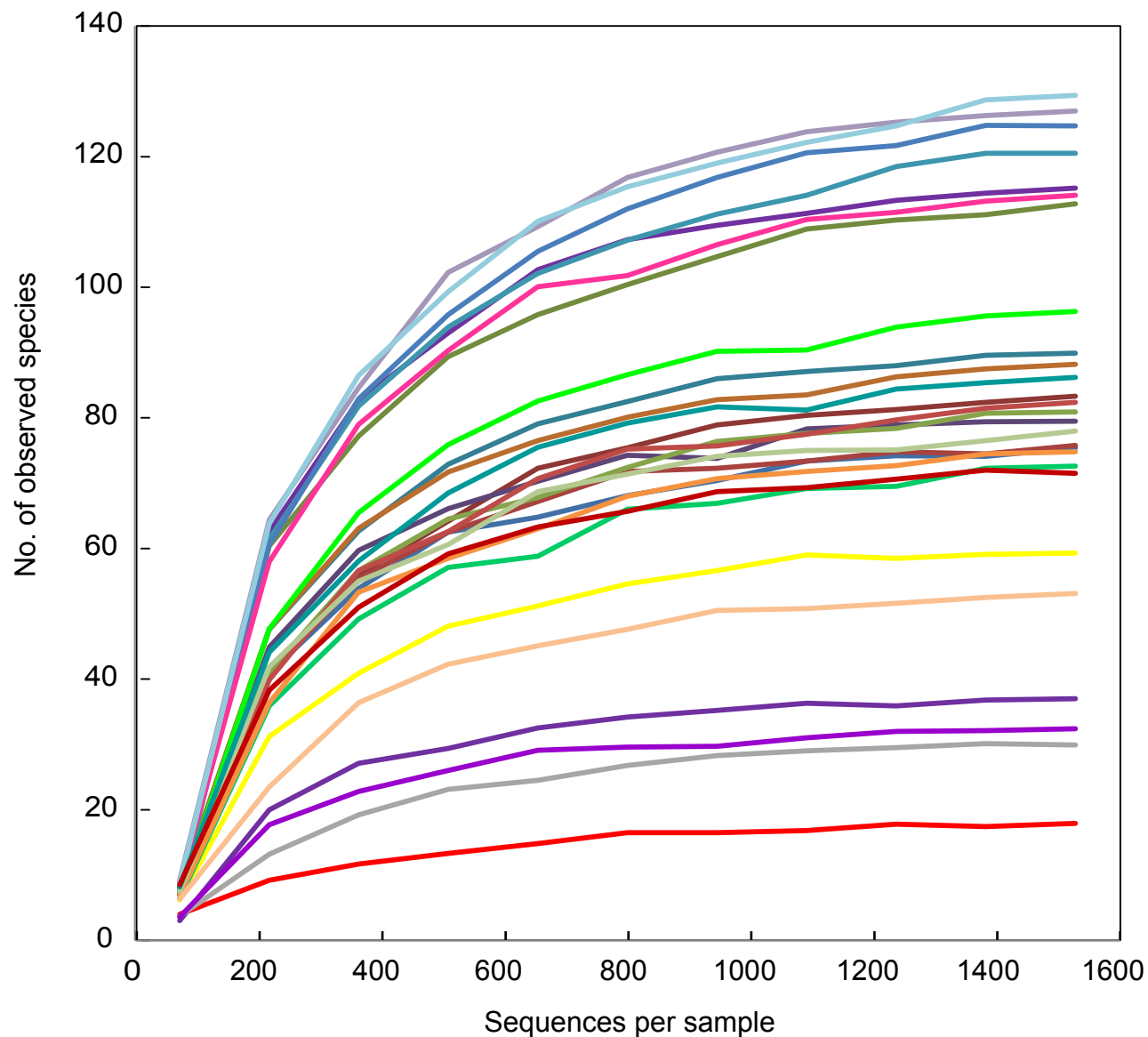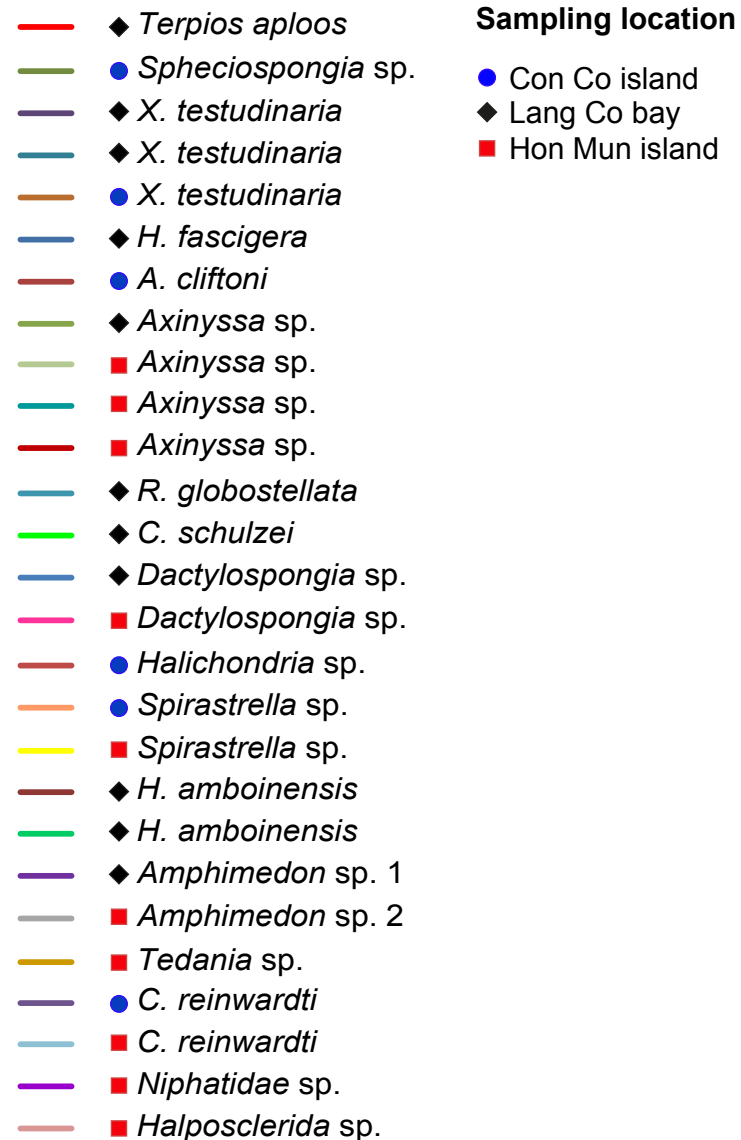

Supplement: Supplemental Information 8 [file peerj-06-4970-s008.pdf]

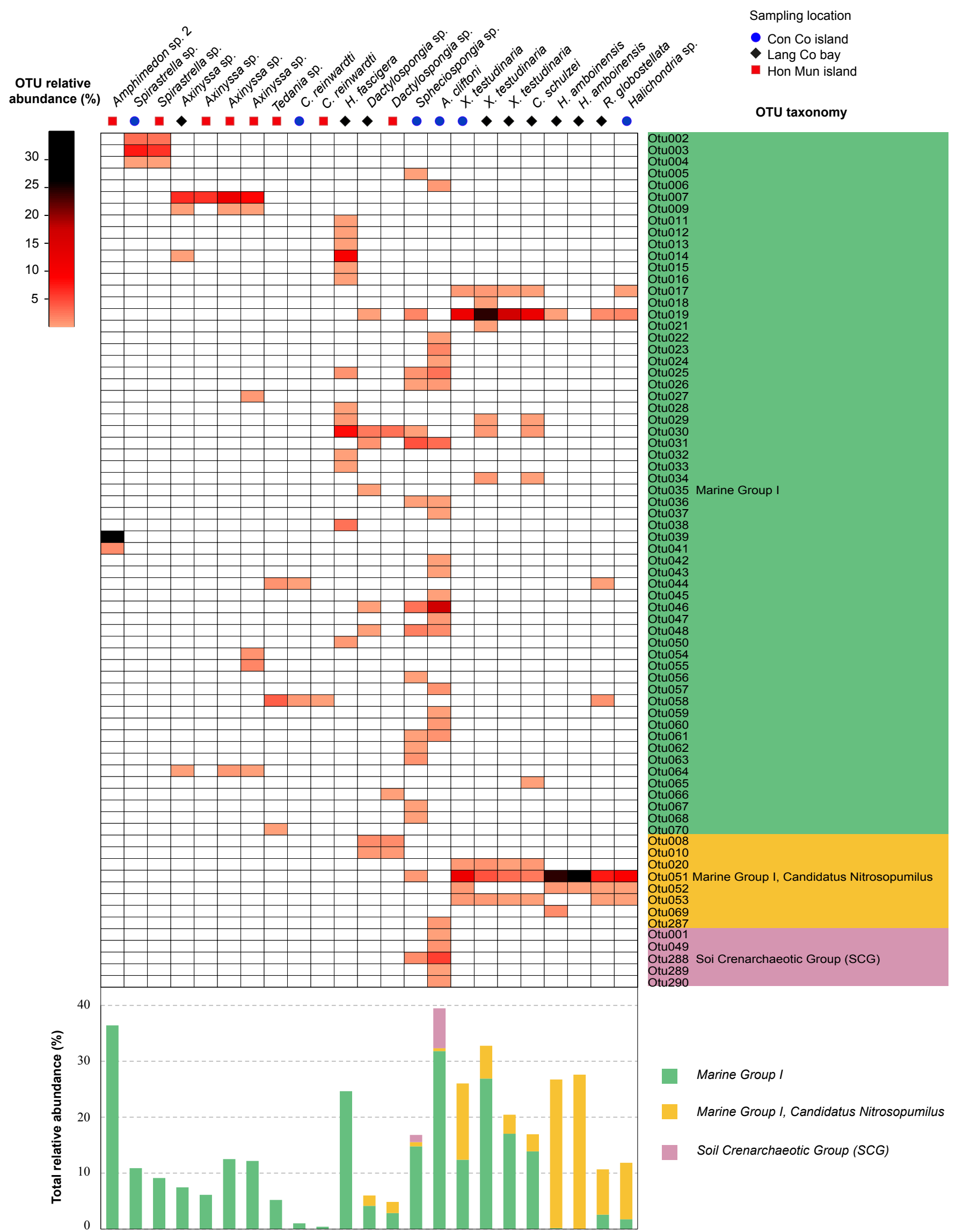

Supplement: Supplemental Information 9 — OTU taxonomy was assigned by NG-tax. [file peerj-06-4970-s009.pdf]

# Sampling location

- Con Co island
- ◆ Lang Co bay
- Hon Mun island

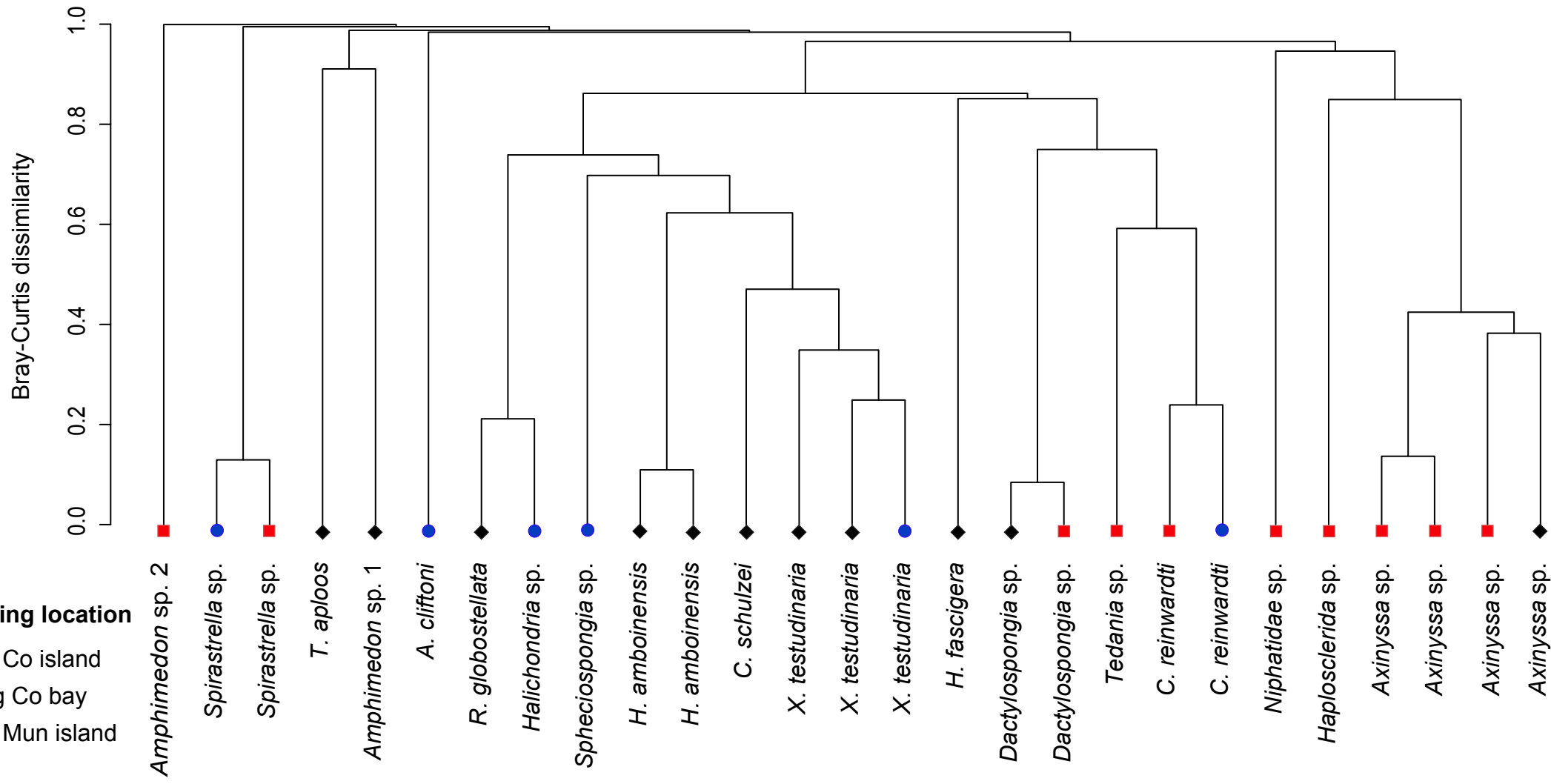

Supplement: Supplemental Information 10 — The hierarchical clustering based on Bray-Curtis similarity was calculated using the vegdist (method = “bray”) function of the vegan package in R version 3.3.1. [file peerj-06-4970-s010.pdf]

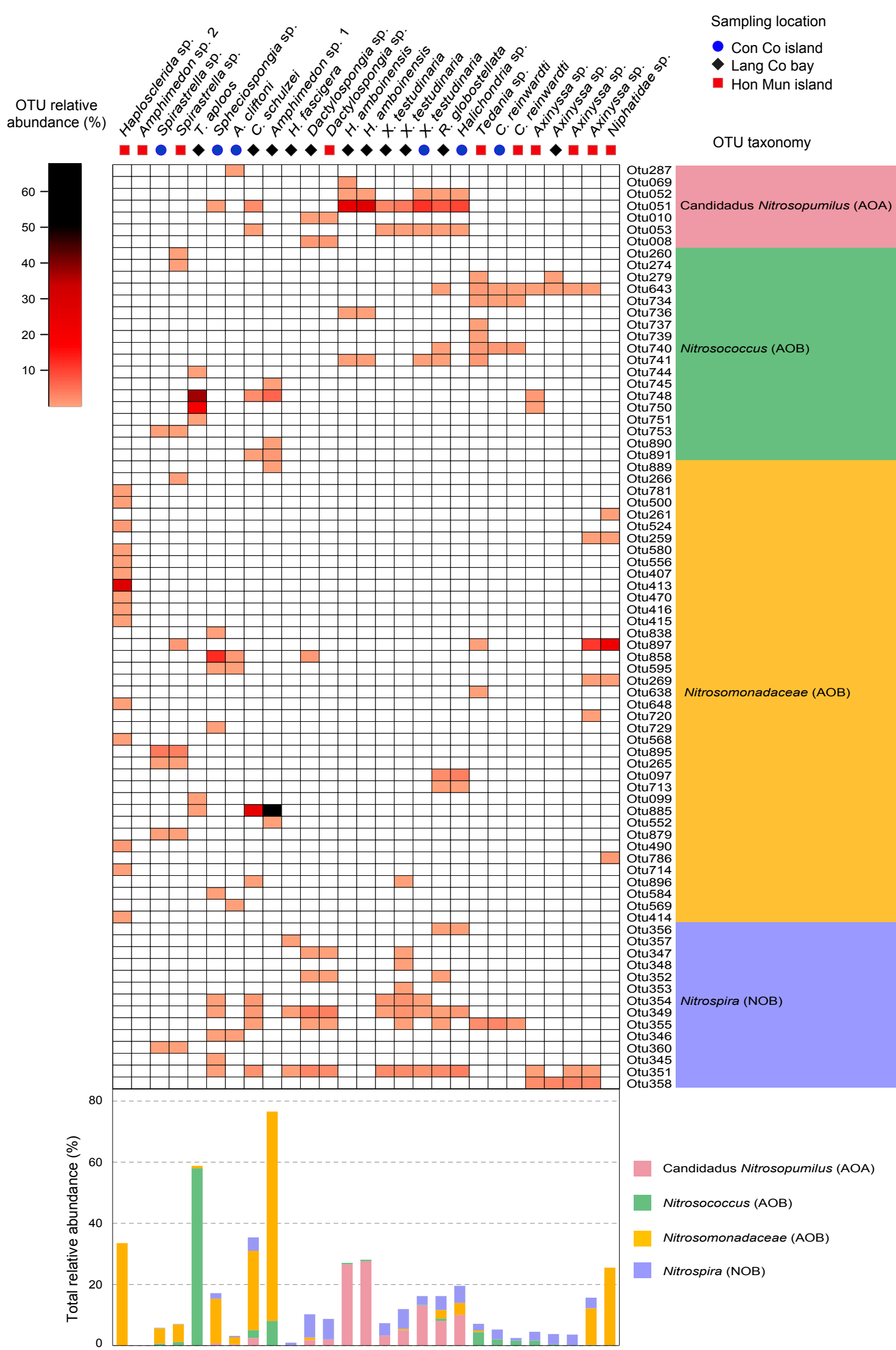

Supplement: Supplemental Information 11 — OTU taxonomy was assigned by NG-tax. [file peerj-06-4970-s011.pdf]
